# Development of novel isatin–nicotinohydrazide hybrids with potent activity against susceptible/resistant *Mycobacterium tuberculosis* and bronchitis causing–bacteria

**DOI:** 10.1080/14756366.2020.1868450

**Published:** 2021-01-07

**Authors:** Zainab M. Elsayed, Wagdy M. Eldehna, Marwa M. Abdel-Aziz, Mahmoud A. El Hassab, Eslam B. Elkaeed, Tarfah Al-Warhi, Hatem A. Abdel-Aziz, Sahar M. Abou-Seri, Eman R. Mohammed

**Affiliations:** a Faculty of Pharmacy, Scientific Research and Innovation Support Unit, Kafrelsheikh University, Kafrelsheikh, Egypt; b Faculty of Pharmacy, Department of Pharmaceutical Chemistry, Kafrelsheikh University, Kafrelsheikh, Egypt; c The Regional Center for Mycology & Biotechnology, Al-Azhar University, Cairo, Egypt; d Department of Pharmaceutical Chemistry, School of Pharmacy, Badr University in Cairo, Badr City, Egypt; e Department of Pharmaceutical Sciences, College of Pharmacy, AlMaarefa University, Ad Diriyah, Saudi Arabia; f Faculty of Pharmacy (Boys), Department of Pharmaceutical Organic Chemistry, Al-Azhar University, Cairo, Egypt; g Department of Chemistry, College of Science, Princess Nourah bint Abdulrahman University, Riyadh, Saudi Arabia; h Department of Applied Organic Chemistry, National Research Center, Giza, Egypt; i Faculty of Pharmacy, Department of Pharmaceutical Chemistry, Cairo University, Cairo, Egypt

**Keywords:** Anti-tubercular activity, DprE1 inhibitors, resistant TB, nicotinohydrazide, isatin derivatives

## Abstract

Joining the global fight against Tuberculosis, the world's most deadly infectious disease, herein we present the design and synthesis of novel isatin-nicotinohydrazide hybrids (**5a–m** and **9a–c**) as promising anti-tubercular and antibacterial agents. The anti-tubercular activity of the target hybrids was evaluated against drug-susceptible M. tuberculosis strain (ATCC 27294) where hybrids **5d**, **5g** and **5h** were found to be as potent as INH with MIC = 0.24 µg/mL, also the activity was evaluated against Isoniazid/Streptomycin resistant M. tuberculosis (ATCC 35823) where compounds **5g** and **5h** showed excellent activity (MIC = 3.9 µg/mL). Moreover, the target hybrids were examined against six bronchitis causing-bacteria. Most derivatives exhibited excellent antibacterial activity. K. pneumonia emerged as the most sensitive strain with MIC range: 0.49–7.81 µg/mL. Furthermore, a molecular docking study has proposed DprE1 as a probable enzymatic target for herein reported isatin-nicotinohydrazide hybrids, and explored the binding interactions within the vicinity of DprE1 active site.

## GRAPHICAL ABSTRACT

**Figure:**
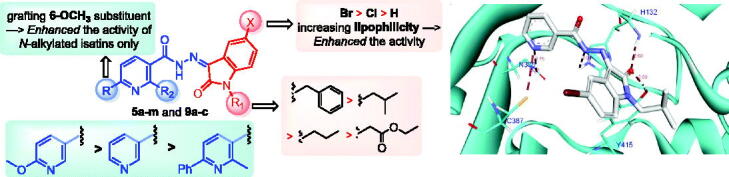

## 1. Introduction

Tuberculosis (TB) is considered to be the world’s top infectious killer^1^. It is a complicated devastating disease that accounts for the death of 1.5 million individuals in 2018 as declared by WHO^1^. About 23% of the world’s population is expected to be latent TB carriers with a high risk to develop the disease^2^. The first-line drugs such as Isoniazid, Rifampicin, Ethambutol, and Streptomycin have shown significant activity against *Mycobacterium tuberculosis* however, the emergency of drug-resistant TB (DR-TB), especially multi-drug resistant TB (MDR-TB) is still a serious trouble^3^. The emerged drug resistance can be considered as a global health crisis, where only one out of three accessing proper remedy among the patients falling ill in 2018^1^.

Along with the emerged drug resistance, the development of secondary bacterial infection is considered to be a critical problem^4^. Different bacterial strains have been isolated from sputum samples including: *Haemophilus influenzae*, *Klebsiella pneumoniae*, *Streptococcus pneumoniae*, and *Moraxella catarrhalis*^4^^,^^5^. The tubercular and the bacterial co-infection can lead to inadequate treatment causing serious complications such as pneumoniae, bronchial anthracofibrosis, and chronic airflow obstruction^5–8^. Unfortunately, many virulent bacterial strains have developed bacterial resistance against most of the currently used antibiotics. The death rate due to bronchitis has been increased up to an alarming level recording about 0.7 million cases of death every year. Thus, there is a crucial need to design and synthesise new candidates that may help in the battle of the developed bacterial resistance towards the currently available anti-tubercular and antibacterial drugs.

**Figure 1.**
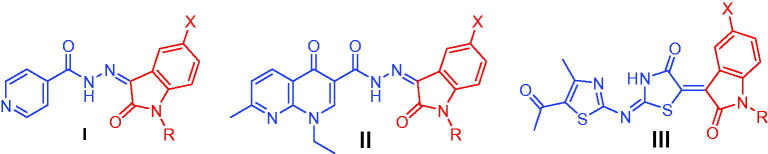
Structures of some reported isatin-based anti-mycobacterial agents (**I–III**).

Isatin is a promising endogenous biologically active scaffold. It could be chemically modified to produce various heterocyclic compounds with different biological activities^9–14^. Isatin based derivatives have revealed promising antibacterial^15–17^ and anti-TB effects^18^^,^^19^. Moreover, the nicotinohydrazide moiety has a large contribution to the field of medicinal chemistry. It has been incorporated in several active antimicrobial^20^ and anti-tubercular agents^21^. In fact, the presence of a hydrazide group can be considered as a significant key for an optimum anti-tubercular activity^22^. Recently, several research teams adopted molecular hybridisation techniques between isatin and different moieties for the design of new anti-mycobacterial agents^23–26^, such as isatin-INH hybrids **I** (Figure 1)^25^ and isatin-nalidixic acid hybrids **II** (Figure 1)^26^. Later, our research team has identified the *N*-substituted isatin-thiazolidinone hybrid **III** (Figure 1) as a promising antitubercular agent that produced an effective killing of *M. aurum* in infected macrophage model with broad-spectrum antimicrobial effect against sensitive and resistant bacterial strains^27^. SAR study revealed that *N*-benzylation or *N*-methylation of the 2-oxindole ring enhanced the activity by 2-4 folds as compared to the *N*-unsubstituted analogues.

**Figure 2.**
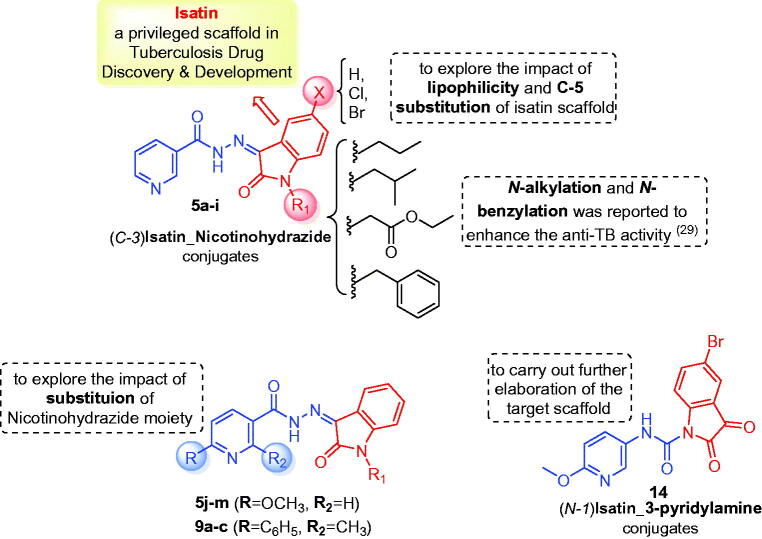
Design of the target hybrids **5a–m**, **9a–c**, and **14**.

In view of the findings stated above and in a continuation of our previous work for the discovery of potential anti-tubercular and anti-microbial agents^28^, we decided to extend our research through conjugating different *N*-substituted isatin moieties with a nicotinohydrazide moiety to furnish target hybrids **5a-I** (Figure 2). In order to explore the impact of C-5 substitution, as well as to ensure different lipophilic environments that may be suitable for the mycobacterial activity, both 5-Cl and 5-Br substituents were grafted at isatin scaffold. As it was suggested that *N*-substitution of isatin scaffold could enhance the mycobacterial activity^27^^,^^29^, *N*-alkylation, or *N*-benzylation of the herein reported hybrids was performed (Figure 2). Thereafter, the impact of C-2 and C-6 substitution of the nicotinohydrazide moiety the anti-tubercular and antibacterial activities were investigated through developing the two sets **5j–m** and **9a–c** (Figure 2). Finally, an isatin-3-pyridylamine hybrid **14** was synthesised to carry out further elaboration of the target scaffold (Figure 2).

Herein seventeen new 2-oxindolin-3-ylidene-nicotinohydrazide derivatives (**5a–m**, **9a–c**, and **14**) were designed, synthesised, and evaluated for their anti-tubercular activity against drug-susceptible *M. tuberculosis* strain (ATCC 27294) and Isoniazid/Streptomycin resistant *M. tuberculosis* (ATCC 35823), together with the antibacterial effect against bronchitis causing-bacteria in an attempt to produce new candidates having potential activities with minimal bacterial resistance that may reduce the complications associated with the secondary bacterial infection.

Finally, molecular docking studies have been performed for the most active compounds into the active sites of two enzymes: flavoenzyme Decaprenylphosphoryl-*β*-D-Ribose 2′-Epimerase (DprE1) and enoyl-acyl carrier protein reductase (InhA) being critical enzymes in the metabolism and synthesis of *M. tuberculosis* cell wall^30^^,^^31^ to explore the possible mechanism of action of the new hybrids.

## 2. Results and discussion

### 2.1. Chemistry

**Scheme 1.**
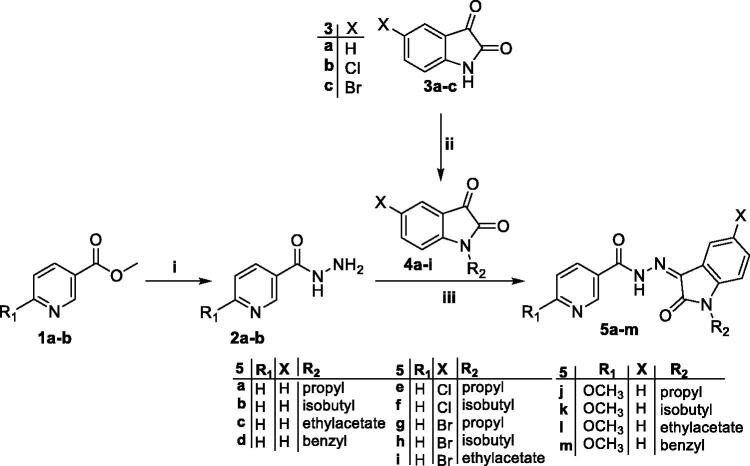
Synthesis of target isatin hybrids **5a–m**; (**i**) NH_2_NH_2_.H_2_O/methanol/reflux 4 h, (**ii**) R_1_-Br/DMF/KI (Cat.)/K_2_CO_3_/reflux 3 h, (**iii**) Ethanol absolute/drops glacial acetic acid (Cat.)/reflux 6 h.

**Scheme 2.**
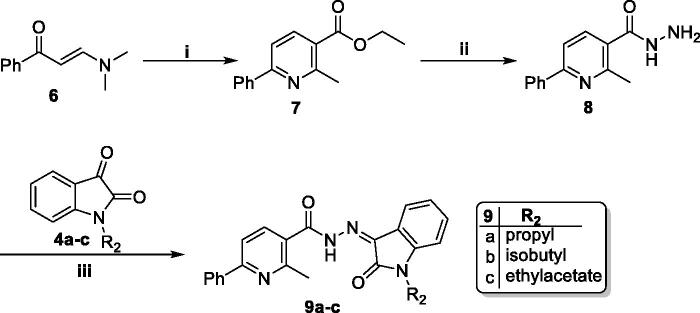
Synthesis of target isatin hybrids **9a–c**; (**i**) ethyl acetoacetate/NH_4_OAc/glacial acetic acid/reflux 6 h, (**ii**) NH_2_NH_2_.H_2_O/methanol/reflux 6 h, (**iii**) Ethanol absolute/drops glacial acetic acid (Cat.)/reflux 6 h.

**Scheme 3.**
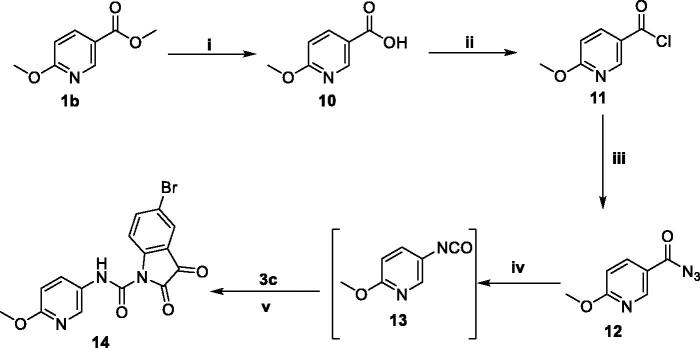
Synthesis of target compound **14**; (**i**) NaOH/MeOH/reflux 6 h, (**ii**) SOCl_2_/reflux 4 h, (**iii**) NaN_3_/acetone/stirring at R.T. 4 h, (**iv**) Dry toluene/reflux 1 h, (**v**) Dry toluene/reflux 5 h.

The synthetic strategies adopted for the preparation of final compounds (**5a–m**, **9a–c** and **14**) were illustrated in Schemes 1–3. In the first scheme, different isatin derivatives **3a–c** were alkylated with the appropriate alkyl halide in anhydrous DMF in the presence of K_2_CO_3_ as an acid binder to furnish the *N*-substituted indoline-2,3-diones **4a–i**. On the other hand, methyl nicotinate **1a** and methyl 6-methoxynicotinate **1b** was refluxed with hydrazine hydrate in methanol to produce the key intermediate nicotinohydrazides **2a–b** which subsequently reacted with *N*-substituted indoline-2,3-dione derivatives **4a–i** in absolute ethanol containing a catalytic amount of glacial acetic acid to get the final compounds **5a–m** (Scheme 1).

In the second scheme, 3-(dimethylamino)-1-phenylprop-2-en-1-one **6** was heated under reflux with ethyl acetoacetate and NH_4_OAc in glacial acetic acid to produce ethyl 2-methyl-6-phenylnicotinate **7** in which, the ester moiety was subjected to hydrazinolysis to produce the key intermediate 2-methyl-6-phenylnicotinohydrazide **8** which reacted with different *N*-substituted indoline-2,3-diones **4a–c** to get target compounds **9a–c**.

In the last scheme, methyl 6-methoxynicotinate **1b** was refluxed with NaOH in MeOH to get the free 6-methoxynicotinic acid **10**, which then undergone to chlorination *via* SOCl_2_ to furnish 6-methoxynicotinoyl chloride **11** where the chloride was subsequently substituted with an azide functionality to produce 6-methoxynicotinoyl azide **12**. The azid derivative **12** was subjected to Curtius Rearrangement to get 5-isocyanato-2-methoxypyridine **13**, that refluxed with 5-bromoindoline-2,3-dione **3c** in dry toluene to furnish the final compound **14**.

^1^H NMR spectra of the final compounds **5a–m** and **9a–c** were confirmed with the presence of a D_2_O exchangeable singlet signal attributable to the proton of the hydrazide linker at *δ* (13.31–13.86) ppm. Moreover, ^1^H NMR spectra for compounds **9a–c** confirmed the presence of singlet signal in the aliphatic region for the CH_3_ group around *δ* 2.71 ppm, in addition to the extra aromatic protons for the 6-phenyl ring at a range of *δ* 7.44–7.57 ppm, whereas, spectra for compounds **5j–m** and **14** confirmed the appearance of singlet signal for OCH_3_ groups at *δ* (3.82–3.97) ppm.

In addition, the propyl moiety in compounds (**5a**, **e**, **g**, **j**, and **9a**) was confirmed with the appearance of two triplet signals for (–CH_2_CH_3_) and (*N*-CH_2_) at *δ* (0.89-0.91) and (3.70–3.75) ppm, respectively. Also, ^1^H NMR spectra for compounds (**5b**, **f**, **h**, **k**, and, **9b**) displayed two doublet signals for (–CH(CH_3_)_2_) and (*N*-CH_2_) in the isobutyl moiety at *δ* (0.90-0.93) and (3.56-3.74) ppm, respectively, whereas, the ethyl acetate group of compounds (**5c**, **i**, **l**, and **9c**) was represented by a triplet signal for (CO-CH_2_CH_3_), quartette signal for (CO–CH_2_CH_3_) as well as singlet signal for (*N*-CH_2_) at *δ* (1.20–1.22), (4.16–4.17) and (4.72–7.74) ppm, respectively. Furthermore, the *N*-benzyl substituted counterparts (**5d** and 5**m**) showed singlet signals around *δ* 5.02 ppm attributed to the benzylic (–CH_2_–C_6_H_5_) protons.

On the other hand, ^13 ^C NMR spectra for hybrids **5a–m** and **9a–c** showed two signals resonating in the range *δ* (161.20–161.84) and (162.90–166.52) ppm attributable for the carbonyl carbons of the nicotinic hydrazide and indoline-2-one moieties, respectively. Moreover, compounds **5c**, **l**, and **9c** showed an extra signal for C = O group for the ester moiety at *δ* 167.86 ppm, whereas, compound **14** displayed the C = O signal for urea linker at *δ* 153.69 ppm, and two signals for indoline-2,3-dione moiety at *δ* 159.57 and 183.65 ppm. Additionally, ^13 ^C NMR spectra for compounds **9a–c** showed a signal corresponding to CH_3_ carbon at *δ* 23.88–27.24 ppm, while, spectra for compounds **5j–m** and **14** displayed a signal corresponding to OCH_3_ carbon at *δ* (53.53–54.44) ppm.

Furthermore, the carbons of the *N*-propyl group in compounds (**5a**, **e**, **g**, **k**, and **9a**) appeared as three signals resonating at *δ* (11.60–11.65 ppm), (20.81–20.87 ppm), and (41.28–41.48 ppm) for –CH_2_CH_3_, *N*-CH_2_CH_2_, and *N*-CH_2_, respectively, and the carbons of the *N*-isobutyl group in compounds (**5f**, **l** and **9b**) appeared as three signals at a range of *δ* (20.36–20.45 ppm), (27.22–23.88 ppm) and (47.10–56.51 ppm) due to –CH(CH_3_)_2_–, –CH(CH_3_)_2_– and –*N*-CH_2_–, respectively. Finally, a signal attributable to the benzylic (–CH_2_–) carbon in the *N*-benzyl derivatives (**5d** and 5**m**) appeared around *δ* 43.10 ppm.

### 2.2. Biological evaluation

#### 2.2.1. Anti-tubercular activity

All of the newly synthesised hybrids were evaluated for their anti-tubercular action towards *M. tuberculosis* (ATCC 27294) using the Microplate Alamar Blue Assay (MABA)^32^. INH was used as the reference drug. The obtained results for the anti-mycobacterial activity were summarised in (Table 1) and expressed as a minimum inhibitory concentration (MIC).

**Table 1.** MIC (µg/mL) for hybrids (**5a–m**, **9a–c**, and **14**) against *M. tuberculosis* (ATCC 27294) and Isoniazid/Streptomycin resistant *M. tuberculosis* (ATCC 35823), and LogP measurements for hybrids (**5a–m**, **9a–c**, and **14**).
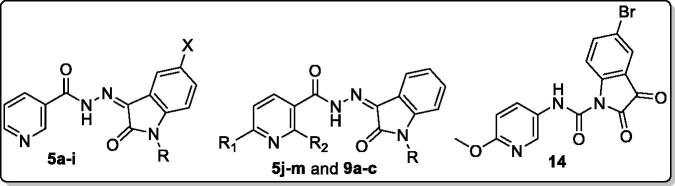

Compound	X or R_2_	R	R_1_	*M. tuberculosis* (ATCC 27294)	*M. tuberculosis* (ATCC 35823)	LogP
**5a**	H	–CH_2_CH_2_CH_3_		3.9	125	1.65
**5b**	H	–CH_2_CH(CH_3_)_2_		1.95	62.5	2.04
**5c**	H	–CH_2_COOCH_2_CH_3_		7.81	NA	1.46
**5d**	H	–CH_2_C_6_H_5_		**0.24**	**15.63**	2.55
**5e**	Cl	–CH_2_CH_2_CH_3_		3.9	NA	2.2
**5f**	Cl	–CH_2_CH(CH_3_)_2_		0.48	**15.63**	2.6
**5g**	Br	–CH_2_CH_2_CH_3_		**0.24**	**3.9**	2.47
**5h**	Br	–CH_2_CH(CH_3_)_2_		**0.24**	**3.9**	2.87
**5i**	Br	–CH_2_COOCH_2_CH_3_		7.81	NA	2.29
**5j**	H	–CH_2_CH_2_CH_3_	OCH_3_	0.98	**15.63**	2.24
**5k**	H	–CH_2_CH(CH_3_)_2_	OCH_3_	0.98	62.5	2.63
**5l**	H	–CH_2_COOCH_2_CH_3_	OCH_3_	3.9	125	2.05
**5m**	H	–CH_2_C_6_H_5_	OCH_3_	7.81	NA	3.14
**9a**	CH_3_	–CH_2_CH_2_CH_3_	C_6_H_5_	31.25	NA	4.45
**9b**	CH_3_	–CH_2_CH(CH_3_)_2_	C_6_H_5_	15.63	NA	4.85
**9c**	CH_3_	–CH_2_COOCH_2_CH_3_	C_6_H_5_	15.63	NA	4.26
**14**				3.9	62.5	1.94
**INH**				0.24	>125	
**Streptomycin**					>125	

NA: not determined, MIC >125 µg/mL.

Bold values represent the best activity.

The results revealed that most tested compounds exerted potent to moderate anti-tubercular action with MIC range = 0.24-7.81 µg/mL. Compounds **5d**, **5g**, and **5h** were found to be as potent as INH with MIC = 0.24 µg/mL. Compounds **5f**, **5j**, and **5k** revealed potent activity with MIC range = 0.48–0.98 µg/mL, whereas derivatives **5a–5c**, **5e**, **5i**, **5l**, **5m**, and **14** exhibited a moderate activity with MIC range = 1.95–7.81 µg/mL.

Concerning the structure–activity relationship, it was observed that the anti-tubercular activity of compounds **5a–5i** was affected by two main factors; *N*-substitution and incorporation of halogen at position 5 of the oxindole ring. Regarding *N*-substitution it was shown that, compounds bearing isobutyl group: **5b**, **5f**, and **5h** exhibited remarkable activity with MIC values = 1.95, 0.48, and 0.24 µg/mL, respectively. Similarly, compound **5d** with *N*-benzyl moiety revealed a potent activity with MIC = 0.24 µg/mL. In contrast, compounds **5c** and **5i** carrying the *N*-ethylcarboxylate group were found to be the least active derivatives (MIC = 7.81 µg/mL). Moreover, the contribution of substitution at position 5 of oxindole ring on the activity was quite important. Incorporating 5-Br group in compounds **5g** and **5h** (MIC = 0.24 µg/mL) showed better activity than 5-Cl substituted compounds **5e** and **5f** (MIC = 0.48 and 3.9 µg/mL, respectively) and the unsubstituted congeners **5a–5c** (MIC range = 3.9–7.81 µg/mL).

Then we turned our attention to study the impact of substitution at position 6 on the nicotinohydrazide moiety of the new hybrids. Comparing the anti-tubercular activity of 6-methoxy nicotinohydrazide derivatives **5j–5m** against the unsubstituted counterparts **5a–5d**, it was found that the introduction of 6-methoxy group in compounds **5j–5l** (MIC = 0.98-3.9 µg/mL) enhanced the anti-tubercular activity by 2-4 fold compared to unsubstituted analogues **5a–5c** (MIC range: 1.95–7.81 µg/mL) except for the benzyl derivatives **5d** and **5m**. Conversely, the introduction of 6-phenyl moiety to the nicotinohydrazide moiety as in compounds **9a–c** (MIC = 15.63–31.25 µg/mL) led to a reduced anti-tubercular activity that may be due to steric factors.

**Figure 3.**
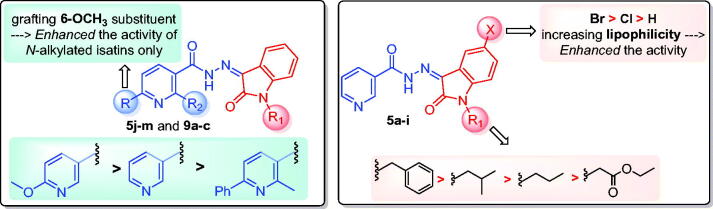
Summary for the structure activity relationships for anti-mycobacterial activity of the target hybrids.

In conclusion, increasing the lipophilicity of the isatin scaffold via C-5 substitution with halogens (Br > Cl) was more advantageous for activity. Also, the anti-mycobacterial action of the target hybrids was decreased in the following order; 6-methoxy nicotinohydrazide > unsubstituted nicotinohydrazide > 2-methyl-6-phenyl nicotinohydrazide (Figure 3).

#### 2.2.2. Anti-tubercular activity towards Isoniazid and Streptomycin resistant M. tuberculosis (ATCC 35823)

The anti-tubercular activity against Isoniazid and Streptomycin resistant *M. tuberculosis* (ATCC 35823) was evaluated using MABA^32^. Investigating the results of the target compounds **5a–m**, **9a–c**, and **14** (Table 1), it was found that many of the tested derivatives exhibited potent to moderate activity with MIC range = 3.9-62.5 µg/mL. Compounds **5g** and **5h** with the best anti-tubercular effect against *M. tuberculosis* (ATCC 27294), showed excellent activity (MIC = 3.9 µg/mL) resistant *M. tuberculosis* strain (ATCC 35823), with more than 32 fold increase in the activity compared to INH and Streptomycin (MIC > 125 µg/mL). Moreover, compounds **5d**, **5f** and **5j** elicited a good activity with MIC = 15.63 µg/mL, whereas derivatives **5b**, **5k**, and **14** produced a moderate activity with MIC = 62.5 µg/mL. Finally, compounds **5a** and **5l** were found to be least active showing MIC value = 125 µg/mL, while the 6-phenyl pyridine derivatives **9a–c** were inactive with MIC >125 µg/mL.

#### 2.2.3. Antibacterial activity against bronchitis causing-bacteria

The antibacterial activity for the synthesised compounds **5a–m**, **9a–c**, and **14** was evaluated against bronchitis causing-bacteria; Gram-negative *Mycoplasma pneumoniae* (ATCC 15531), *K. pneumoniae* (ATCC 43816), *H. influenzae* (ATCC 10211), *M. catarrhalis* (ATCC 25238), *B. pertussis* (ATCC 9340) and Gram-positive *S. pneumoniae* (ATCC 1659) using XTT Susceptibility Assay (Table 2)^33^^,^^34^. The results showed that, most of the evaluated compounds produced a significant antibacterial activity in comparison to the reference drug Azithromycin against the tested bacterial strains. The most sensitive strain towards the new hybrids was *K. pneumoniae* (ATCC 43816) with MIC range = 0.49–7.81 µg/mL, while *M. pneumoniae* (ATCC 15531) and *M. catarrhalis* (ATCC 25238) were the least sensitive strains MIC range = 0.98–125 µg/mL and MIC range = 3.9 to >125 µg/mL, respectively.

**Table 2.** MIC (µg/mL) for hybrids (**5a–m**, **9a–c**, and **14)** against bronchitis causing-bacteria as determined using XTT assay.

Compound	*M. pneumoniae* (ATCC 15531)	*H. influenzae* (ATCC 10211)	*S. pneumoniae* (ATCC 1659)	*K. pneumoniae* (ATCC 43816)	*M. catarrhalis* (ATCC 25238)	*B. pertussis* (ATCC 9340)
**5a**	7.81	7.81	1.95	**0.49**	31.25	3.9
**5b**	1.95	**0.98**	1.95	**0.49**	7.81	1.95
**5c**	31.25	7.81	3.9	1.95	NA	7.81
**5d**	3.9	0.98	1.95	**0.49**	**7.81**	1.95
**5e**	7.81	7.81	1.95	0.98	15.63	7.81
**5f**	3.9	**1.95**	1.95	**0.49**	**3.9**	**1.95**
**5g**	**1.95**	**0.98**	1.95	**0.24**	**3.9**	**1.95**
**5h**	**1.95**	**0.98**	1.95	**0.49**	**3.9**	**1.95**
**5i**	15.63	7.81	1.95	0.98	NA	7.81
**5j**	3.9	3.9	1.95	**0.49**	**7.81**	**3.9**
**5k**	**0.98**	**0.98**	1.95	**0.49**	3.9	**1.95**
**5l**	7.81	15.63	1.95	0.98	15.63	15.63
**5m**	62.5	7.81	7.81	1.95	NA	7.81
**9a**	125	31.25	7.81	7.81	NA	15.63
**9b**	125	62.5	15.63	7.81	NA	31.25
**9c**	125	15.63	15.63	3.9	NA	7.81
**14**	3.9	**1.95**	1.95	**0.49**	15.63	**1.95**
**Azith.**	1.95	3.9	0.98	0.49	7.81	3.9

NA: not determined.

Bold values represent the best activity.

Interestingly, compounds **5g** and **5h** showing best anti-tubercular activity with the minimal bacterial resistance against resistant-TB, revealed a broad-spectrum antibacterial action against most of the tested bronchitis causing-bacteria. They were found to be more potent than the reference drug against *H. influenzae*, *M. catarrhalis*, and *B*. *pertussis* (MIC = 0.24–3.9 µg/mL, Azithromycin MIC = 3.9–7.81 µg/mL). Additionally, they were equipotent to Azithromycin against *M. pneumoniae, S. pneumoniae* and *K. pneumoniae* (MIC = 1.95, 0.98, and 0.49 µg/mL, respectively).

Moreover, compound **5k** showed MIC values (0.98-3.9 µg/mL) less than that expressed by Azithromycin (MIC = 1.95–7.81 µg/mL) against four bacterial strains: *M. pneumoniae* (ATCC 15531), *H. influenza*, *M. catarrhalis* (ATCC 25238) and *B. pertussis* (ATCC 9340). Also, compound **5j** was as potent as the reference drug against *H. influenzae, K. pneumoniae* (ATCC 43816), *M. catarrhalis* (ATCC 25238), and *B. pertussis* (ATCC 9340). Furthermore, compounds **5f** and **14** were two-fold more potent (with MIC = 1.95 µg/mL) than Azithromycin (MIC = 3.9 µg/mL) against *H. influenza* (ATCC 10211) and *B. pertussis* (ATCC 9340) and equipotent to Azithromycin against *K. pneumoniae* (ATCC 43816) with MIC = 0.49 µg/mL. Finally, derivatives **5a**, **5b**, and **5d** produced the same MIC values = 0.49 µg/mL as that expressed by the standard drug against *K. pneumoniae* (ATCC 43816).

#### 2.2.4. Cytotoxicity against non-tumorigenic lung fibroblast WI-38 cells

In order to assess the cytotoxic impact of hybrids **5g** and **5h** on normal human cells, an MTT cytotoxicity assay was carried out against non-tumorigenic WI-38 cells. The obtained results ascribed to hybrids **5g** and **5h** non-significant cytotoxic action with IC_50_ value = 60.95 and 54.01, respectively (Table 3), thus providing favourable selectivity indices (SI) equal to 15.6 and 13.8, respectively.

**Table 3.** *In vitro* cytotoxic effect for hybrids **5g** and **5h** towards non-tumorigenic WI-38 cells, and their Selectivity index (S.I.).

Compound	IC_50 _WI-38 cells (µg/mL)	MIC Resistant TB (µg/mL)	Selectivity Index
**5g**	60.95 ± 3.17	3.9	15.6
**5h**	54.01 ± 2.69	3.9	13.8

## 3. Molecular docking

Over the past decade, several druggable targets have been utilised in TB-drug discovery to develop new therapies in order to fight the global epidemic of TB. Most of these enzyme targets have an essential role in energy production, cell wall biosynthesis, and DNA replication, repair, and transcription. Examples of these enzymes are; Alanine racemase (Alr), Maltosyltransferase (GlgE), *N*-Acetylglucosamine-1-phosphate uridyltransferase (GlmU), Pantothenate synthetase (PS), Mtb ATP synthase subunit C (AtpE), Flavin-dependent thymidylate synthase (TS or ThyA), and flavoenzyme Decaprenylphosphoryl-*β*-D-Ribose 2′-Epimerase (DprE1)^30^.

On account of their superior anti-mycobacterial activity, compounds **5g** and **5h** were selected for further *in silico* investigation to explore their possible molecular target. Two promising drug enzymes in *M. tuberculosis* were suggested as potential targets for the target compounds in this study; the flavoenzyme Decaprenylphosphoryl-*β*-D-Ribose 2′-Epimerase (DprE1) and the enoyl-acyl carrier protein reductase (InhA) enzymes. Both enzymes play an essential role in the metabolism of the cell wall and the synthesis of mycolic acid, respectively^30^^,^^31^. The selection of these two enzymes is based on a pilot docking study that explored the plausible binding interactions and energy scores for the prepared compounds with different enzyme targets.

The crystal structures of the two enzymes were downloaded from the protein data bank PDB ID: 4NCR for DprE1 and 2B35 for InhA. Despite, the importance of docking in providing a predicated binding mode and an account for a ligand strength, it remains a theoretical technique that needs proper validation by comparison with an experimental reference. Therefore, both co-crystallised ligands were re-docked into their corresponding enzymes. The calculated RMSD values between the co-crystallised and docked pose were 0.52 and 0.71 for DprE1 and InhA, orderly which indicates a valid and reliable docking protocol. Compound **5h** has achieved −10.1 and −7.9 kcal/mole energy score for DprE1 and InhA, respectively, whereas, compound **5g** has achieved −9.7 and −7.3 kcal/mole for DprE1 and InhA, respectively. Interestingly, both compounds **5g** and **5h** achieved a higher energy score than the co-crystalized ligand (−9.3 kcal/mole) in DprE1. These findings suggested that DprE1 may be a plausible target for the herein reported compounds.

**Figure 4.**
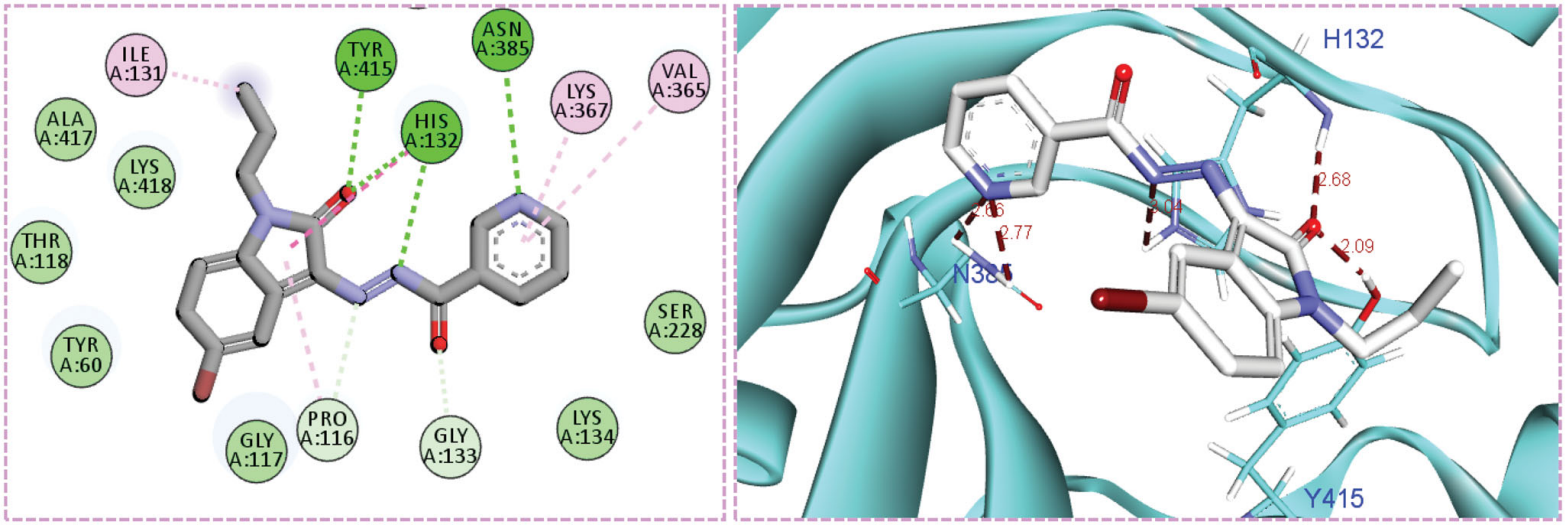
The 2D diagram and 3D representation for compound **5g** displaying its interactions with the DprE1 binding site.

**Figure 5.**
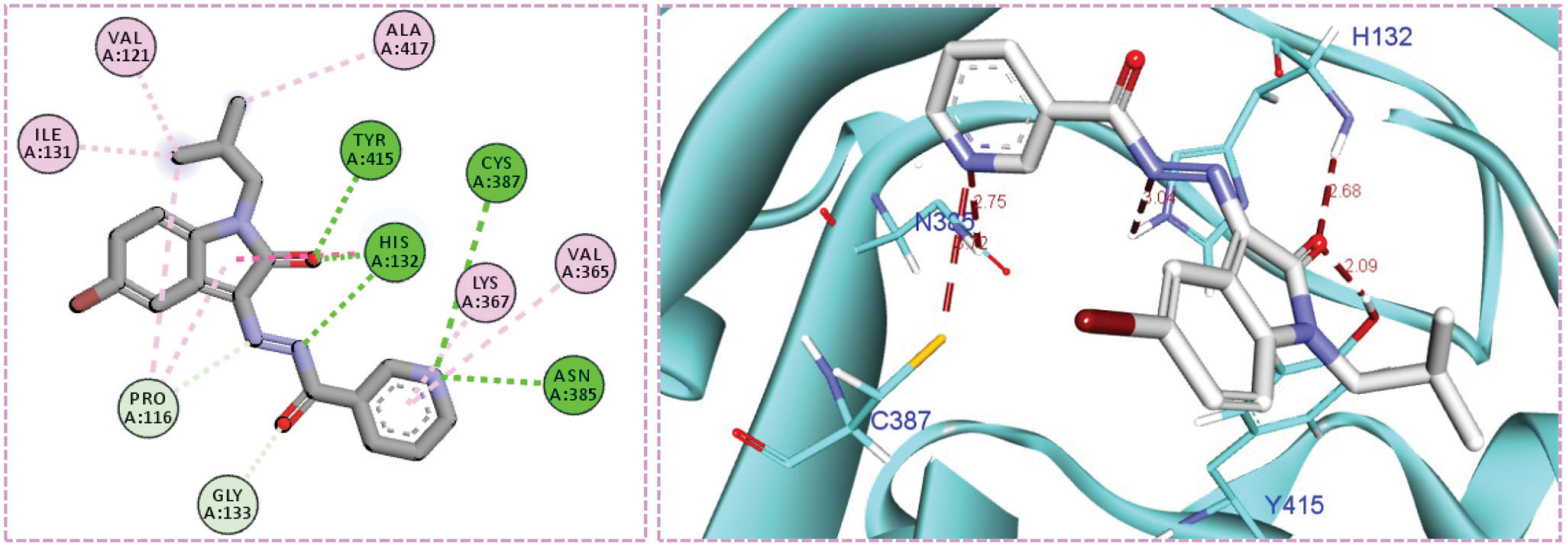
The 2D diagram and 3D representation for compound **5h** displaying its interactions with the DprE1 binding site.

Inspection of the docking poses for the two examined compounds revealed their ability to fit perfectly in the active site of the DprE1 enzyme establishing different types of interactions (Figures 4 and 5). The oxindole ring in both compounds **5g** and **5h** was engaged in two H-bond interactions *via* its carbonyl oxygen (C = O) functionality with His-132 and Tyr-415 amino acids. Furthermore, the NH of the hydrazide linker and the nitrogen atom of the pyridine ring were involved in H-bond interactions with His-132 and Asn-385, respectively. Moreover, the nitrogen atom of the pyridine ring in compound **5h** achieved an extra hydrogen bond interaction with Cys-387 which may explain its higher energy score than **5g**. Worthy of note, the *N*-isobutyl group of compound **5h** established several hydrophobic interactions with non-polar residues lining a hydrophobic pocket, such as Pro-116, Val-121, Ile-131, and Ala-417.

The 2 D diagrams for interactions of compounds **5g** and **5h** in InhA active site (Figure S1), and the 3 D illustrations of the superimposition for the docking poses and the co-crystalized ligands in DprE1 and InhA active sites (Figure S2), as well as the detailed interactions achieved by compounds **5g** and **5h** within the DprE1 binding site (Table S3) were provided in the Supporting Materials.

## 4. Conclusion

In this work, different sets of isatin-nicotinohydrazide hybrids (**5a–m**, **9a–c**) and isatin-3-pyridylamine hybrid **14** were designed and synthesised. The anti-mycobacterial activity towards *M. tuberculosis* (ATCC 27294), as well as towards the INH and Streptomycin resistant *M. tuberculosis* (ATCC 35823), were evaluated for the seventeen newly synthesised 2-oxindolin-3-ylidene-nicotinohydrazide derivatives. The obtained results identified compounds **5g** and **5h** as the most potent anti-tubercular agents in this study (MIC = 0.24 µg/mL), with minimal mycobacterial resistance (MIC = 3.9 µg/mL). Furthermore, all herein reported hybrids were examined for their antibacterial activity against six bronchitis causing-bacteria; *M. pneumoniae*, *H. influenzae*, *K. pneumoniae*, *M. catarrhalis*, *B. pertussis*, and *S. pneumonia*. Most hybrids exerted significant antibacterial activity in comparison to the reference drug Azithromycin against the tested bacterial strains. The most sensitive strain was *K. pneumoniae* with MIC range = 0.49–7.81 µg/mL. Interestingly, compounds **5g** and **5h** displayed a broad-spectrum antibacterial activity against most of the tested bronchitis causing-bacteria. Moreover, an *in silico* molecular modelling study has proposed DprE1 as a potential enzyme target for herein reported isatin-nicotinohydrazide hybrids. Compounds **5g** and **5h** were able to fit perfectly in the active site of the DprE1 enzyme achieving a higher energy score (−9.7 and −10.1 kcal/mole, respectively) than the co-crystalized ligand (−9.3 kcal/mole). The oxindole ring was engaged in two H-bond interactions via its carbonyl oxygen (C = O) functionality with His-132 and Tyr-415 amino acids, whereas, the NH of the hydrazide linker and the nitrogen atom of the pyridine ring were involved in H-bond interactions with His-132 and Asn-385, respectively. In addition, the *N*-isobutyl group of compound **5h** established several hydrophobic interactions with non-polar residues lining a hydrophobic pocket.

## 5. Experimental

### 5.1. Chemistry

#### 5.1.1. General

I.R. spectra have been recorded on Schimadzu FT-IR 8400S spectrophotometer. NMR spectra were recorded by Bruker-Avance 400 spectrometer. ^13 ^C NMR spectra were run at 100 MHz in deuterated dimethylsulphoxide (DMSO-d_6_). Chemical shifts (*δ_H_*) are reported relative to the solvent (DMSO-d_6_*)*. NMR analyses were carried out at the NMR Unit, Faculty of Pharmacy, Mansoura University, Mansoura, Egypt, whereas IR analyses were carried out at the Faculty of Science, Mansoura University, Mansoura, Egypt. Elemental analyses were carried out at the Regional Centre for Microbiology and Biotechnology, Al-Azhar University, Cairo, Egypt. Compounds **2a**,**b**^35^, **4a–i**^36^^,^^37^, **8**^38^, and **11**^39^, were prepared as described previously.

#### 5.1.2. Synthesis of target compounds 5a–m and 9a–c

In a round-bottom flask, the appropriate nicotinohydrazide derivative **2a–b** or **8** (2.5 mmol) were added to an equivalent amount of *N*-substituted indoline-2,3-dione derivatives **4a–i** in 15 ml of glacial acetic acid. The previous mixture was heated under reflux with TLC monitoring, after complete consummation of starting materials, the reaction flask was cooled to room temperature. The formed precipitate was collected under vacuum, washed with hexane, cold water and recrystallized from dioxane/methanol mixture to furnish final compounds **5a–m** and **9a–c**, respectively.

#### 5.1.3. Synthesis of target compound 14

6-Methoxynicotinoyl azide **12** (0.3 g, 1.6 mmol) was dissolved in anhydrous toluene (20 ml) which then heated under reflux for 1 h. To the previous solution, an equivalent amount of 5-bromoisatin was added and the reaction mixture was refluxed for 5 h. The produced precipitate was filtrated while hot washed with hexane and recrystallized from acetonitrile to furnish compound **14**.

Full characterisation details (NMR, IR, and elemental analysis) for the target hybrids (**5a–m**, **9a–c**, and **14**) have been provided in the Supporting Materials.

### 5.2. Biological evaluation

#### 5.2.1. Anti-tubercular activity

Microplate Alamar blue assay (MABA)^32^ has been utilised to determine MICs for all herein prepared hybrids (**5a–m**, **9a–c**, and **14**) against M. tuberculosis ATCC 27294 (Isoniazid-susceptible strain) and *Mycobacterium tuberculosis* ATCC 35823 (Isoniazid and Streptomycin resistant strain). The detailed procedures were presented in the supporting materials.

#### 5.2.2. XTT Susceptibility Assay for the antibacterial activity

A colorimetric broth microdilution method was utilised to determine the MIC for the tested hybrids following the XTT [2,3-bis(2-methoxy-4-nitro-5-sulfo-phenyl)-2H-tetrazolium-5-carboxanilide]-reduction assay^33^^,^^34^. MICs for the tested hybrids was assessed against bacteria causing bronchitis; Gram-negative bacteria: *H. influenzae ATCC 10211*, *M. pneumoniae ATCC 15531*, *K. pneumoniae ATCC 43816, M. catarrhalis ATCC 25238*, and *Bordetella pertussis ATCC 9340*, as well as *S. pneumoniae ATCC 1659*, representing Gram-positive bacterium, that obtained from the American type culture collection. The detailed experimental procedures have been added in the supporting materials.

#### 5.2.3. Cytotoxicity assay

The MTT assay^40^ was used to assess the cytotoxic impact of hybrids **5g** and **5h** towards the normal human lung WI-38 cells as reported earlier^41^.

### 5.3. Molecular docking

Aiming to define the molecular target for the best two lead compounds, the crystal structures of both DprE1 and InhA enzymes of TB were downloaded from the Protein data bank ID: 4NCR and 2B35, respectively^42^^,^^43^. Vina Autodock software was implemented in the current work to conduct the docking studies. The program demands both the ligand and the receptor in the pdbqt format, for that, M.G.L tools were used to prepare the two enzymes and the ligands into the right format^44^. Also, the active sites were determined by generating a grid box sized 24 × 24 × 24 Å surrounding the binding domain of each co-crystallised ligand with its corresponding enzyme (centre_x = 17.3, centre_y = −20 and centre_z = 3.4 for 4ncr and centre_x = 17, centre_y = 12.5 and centre_z = 10.5 for 2b35). Docking validity and reliability were ensured by re-docking each co-crystallised ligands with their corresponding enzyme, then calculating the RMSD between the co-crystallised pose and the docked pose. Discovery studio 4.5 visualiser was employed in the analysis of docking results as well as in the evaluation of best candidates based on binding affinity score and interaction with receptor^45^.

## Supplementary Material

Supplemental Material
