# Supplementary material for: Development of novel isatin–nicotinohydrazide hybrids with potent activity against susceptible/resistant Mycobacterium tuberculosis and bronchitis causing–bacteria
Source: J Enzyme Inhib Med Chem. 2021 Jan 7;36(1):384–92. doi: 10.1080/14756366.2020.1868450 (PMC7801109; doi:10.1080/14756366.2020.1868450)
Supplement: Supplemental Material [file IENZ_A_1868450_SM7310.pdf]

Supporting Information

Development of novel isatin-nicotinohydrazide hybrids with potent activity against susceptible/resistant Mycobacterium tuberculosis and bronchitis causing-bacteria

Zainab M. Elsayed, Wagdy M. Eldehna*, Marwa M. Abdel-Aziz, Mahmoud A. El Hassab, Eslam B. Elkaeed, Tarfah Al-Warhi, Hatem A. Abdel-Aziz, Sahar M. Abou-Seri, Eman R. Mohammed

1. Characterisation details (NMR, IR and elemental analysis) for the target hybrids (5a-m, 9a-c and 14)

N'-(2-Oxo-1-propylindolin-3-ylidene)nicotinohydrazide ***5a***.

Yellow powder, m.p. 141-143 °C; (yield 81%); ^{1}H NMR *δ ppm*: 0.90 (3H, t, *J*= 8.0 Hz, -$CH_2C\underline{H_3}$), 1.63-1.73 (2H, m, *N*-$CH_2C\underline{H_2}$), 3.73 (2H, t, *J*= 8.0 Hz, *N*-CH_2), 7.16 (1H, t, *J*= 8.0 Hz, H-5 of nicotinic hydrazide), 7.24 (1H, d, *J*= 8.0 Hz, H-7 of 2-indolinone), 7.46 (1H, t, *J*= 8.0 Hz, H-6 of 2-indolinone), 7.60-7.67 (2H, m, H-5 and H-4 of 2-indolinone), 8.25 (1H, d, *J*= 8.0 Hz, H-4 of nicotinic hydrazide), 8.84 (1H, d, *J*= 8.0 Hz, H-6 of nicotinic hydrazide), 9.07 (1H, s, H-2 of nicotinic hydrazide), 13.86 (1H, s, NH); ^{13}C NMR *δ ppm*: 11.64 (-$CH_2\underline{C}H_3$), 20.86 (*N*-$CH_2\underline{C}H_2$), 41.32 (*N*-CH_2), 110.77, 119.44, 121.37, 123.75, 124.56, 128.42, 132.46, 135.95, 143.65, 148.98, 153.63, 161.56 (C=O nicotinic hydrazide), 163.78 (C=O 2-indolinone); IR (KBr, *ν* cm^{-1}) 3452 (NH) and 1706, 1678 (2C=O); Analysis calculated for $C_{17}H_{16}N_4O_2$: , 66.22; H, 5.23; N, 18.17; found , 66.45; H, 5.26; N, 18.24.

N'-(1-Isobutyl-2-oxindolin-3-ylidene)nicotinohydrazide ***5b***.

Yellow powder, m.p. 150-152 °C; (yield 76%); ^{1}H NMR *δ ppm*: 0.93 (6H, d, *J*= 6.8 Hz, -$CH(C\underline{H_3})_2$), 2.07-2.14 (1H, m, -$C\underline{H}(CH_3)_2$), 3.58 (2H, d, *J*= 8.0 Hz, *N*-CH_2), 7.16 (1H, t, *J*= 7.6 Hz, H-5 of nicotinic hydrazide), 7.23 (1H, d, *J*= 8.0 Hz, H-7 of indoline-2,3-dione), 7.46 (1H, t, *J*= 8.0 Hz, H-6 of 2-indolinone), 7.62-7.67 (2H, m, H-5 and H-4 of 2-indolinone), 8.25 (1H, d, *J*= 8.0 Hz, H-4 of nicotinohydrazide), 8.84 (1H, d, *J*= 5.6 Hz, H-6 of nicotinic hydrazide), 9.07 (1H, s, H-2 of nicotinic hydrazide), 13.84 (1H, s, NH); IR (KBr, *ν* cm^{-1}) 3369 (NH) and 1697, 1680 (2C=O); Analysis calculated for $C_{18}H_{18}N_4O_2$: C, 67.07; H, 5.63; N, 17.38; found C, 66.82; H, 5.58; N, 17.96.

Ethyl-2-(3-(2-nicotinoylhydrazono)-2-oxindolin-1-yl)acetate ***5c***.

Yellow powder, m.p. 158-160 °C; yield 69%; ^{1}H NMR *δ ppm*: 1.20 (3H, t, *J*= 8.0 Hz, -$CH_2C\underline{H_3}$), 4.16 (2H, q, *J*= 8.0 Hz, -$C\underline{H_2}CH_3$), 4.74 (2H, s, *N*-CH_2), 7.20-7.24 (2H, m, H-5 of nicotinic hydrazide and H-7 of 2-indolinone), 7.48 (1H, t, *J*= 8.0 Hz, H-6 of 2-indolinone), 7.64-7.69 (2H, m, H-5 and H-4 of 2-indolinone), 8.27 (1H, d, *J*= 8.0 Hz, H-4 of nicotinic hydrazide), 8.84 (1H, brs, H-6 of nicotinic hydrazide), 9.08 (1H, s, H-2 of nicotinic hydrazide), 13.59 (1H, s, NH); ^{13}C NMR *δ ppm*: 14.47 (-$CH_2\underline{C}H_3$), 41.42 (-$\underline{C}H_2CH_3$), 61.95 (*N*-CH_2), 110.91, 119.30,

121.42, 124.18, 124.53, 128.40, 132.49, 136.10, 143.31, 148.91, 153.67, 161.46 (C=O nicotinic hydrazide), 163.15 (C=O 2-indolinone), 167.88 (C=O ester); IR (KBr, ν cm^{-1}) 3237 (NH) and 1742, 1710, 1686 (3C=O); Analysis calculated for $C_{18}H_{16}N_4O_4$: C, 61.36; H, 4.58; N, 15.90; found C, 61.51; H, 4.63; N, 15.87.

N'-(1-Benzyl-2-oxindolin-3-ylidene)nicotinohydrazide ***5d***.

Yellow powder , m.p. 167-169 °C; yield 77%; ^{1}H NMR δ *ppm*: 5.04 (2H, s, benzylic protons), 7.09 (1H, d, *J*= 8.0 Hz, H-7 of 2-indolinone), 7.16 (1H, t, *J*= 7.6 Hz, H-5 of nicotinic hydrazide), 7.28 (1H, t, *J*= 8.0 Hz, H-6 of 2-indolinone), 7.34 (2H, t, *J*= 8.0 Hz, H-3 and H-5 of benzyl moiety), 7.41-7.45 (3H, m, H-2, H-4 and H-6 of benzyl moiety), 7.65-7.68 (2H, m, H-5 and H-4 of 2-indolinone), 8.29 (1H, d, *J*= 8.0 Hz, H-4 of nicotinic hydrazide), 8.85 (1H, d, *J*= 5.6 Hz, H-6 of nicotinic hydrazide), 9.10 (1H, s, H-2 of nicotinic hydrazide), 13.80 (1H, s, NH); ^{13}C NMR δ *ppm*: 43.10 (benzylic carbon), 111.09, 119.65, 121.43, 123.98, 124.57, 127.93, 128.16, 128.48, 129.21, 132.36, 136.04, 139.03, 143.32, 149.05, 153.67, 161.58 (C=O nicotinic hydrazide), 163.47 (C=O 2-indolinone); IR (KBr, ν cm^{-1}) 3449 (NH) and 1699, 1680 (2C=O); Analysis calculated for $C_{21}H_{16}N_4O_2$: C, 70.77; H, 4.53; N, 15.72; found C, 70.49; H, 4.55; N, 15.79.

N'-(5-Chloro-2-oxo-1-propylindolin-3-ylidene)nicotinohydrazide ***5e***.

Orange powder, m.p. 151-153 °C; yield 82%; ^{1}H NMR δ *ppm*: 0.90 (3H, t, *J*= 8.0 Hz, -$CH_2\underline{H_3}$), 1.62-1.71 (2H, m, *N*-$CH_2C\underline{H_2}$), 3.73 (2H, t, *J*= 8.0 Hz, *N*-CH_2), 7.30 (1H, d, *J*= 8.0 Hz, H-7 of 2-indolinone), 7.53 (1H, d, *J*= 8.0 Hz, H-6 of 2-indolinone), 7.62 (1H, s, H-4 of of 2-indolinone), 7.65 (1H, t, *J*= 8.0 Hz, H-5 of nicotinic hydrazide), 8.27 (1H, d, *J*= 8.0 Hz, H-4 of nicotinic hydrazide), 8.85 (1H, d, *J*= 8.0 Hz, H-6 of nicotinic hydrazide), 9.08 (1H, s, H-2 of nicotinic hydrazide), 13.75 (1H, s, NH); ^{13}C NMR δ *ppm*: 11.60 (-$CH_2\underline{C}H_3$), 20.81 (*N*-$CH_2\underline{C}H_2$), 41.48 (*N*-CH_2), 112.41, 120.88, 121.24, 124.57, 127.95, 128.31, 131.74, 136.16, 142.35, 143.79, 149.11, 153.74, 161.33 (C=O nicotinic hydrazide), 163.16 (C=O 2-indolinone); IR (KBr, ν cm^{-1}) 3450 (NH) and 1704, 1682 (2C=O); Analysis calculated for $C_{17}H_{15}ClN_4O_2$: C, 59.57; H, 4.41; N, 16.35;found C, 59.78; H, 4.43; N, 16.41.

N'-(5-Chloro-1-isobutyl-2-oxindolin-3-ylidene)nicotinohydrazide ***5f***.

Orange powder, m.p. 172-174 °C; yield 65%; ^{1}H NMR δ *ppm*: 0.91 (6H, d, *J*= 6.8 Hz, -$CH(C\underline{H_3})_2$), 1.89-2.11 (1H, m, -$C\underline{H}(CH_3)_2$), 3.59 (2H, d, *J*= 8.0 Hz, *N*-CH_2), 7.27 (1H, d, *J*= 8.0

Hz, H-7 of 2-indolinone), 7.51 (1H, d, *J*= 8.0 Hz, H-6 of 2-indolinone), 7.63-7.67 (2H, m, H-4 and H-5 of of 2-indolinone), 8.26 (1H, d, *J*= 8.0 Hz, H-4 of nicotinic hydrazide), 8.84 (1H, d, *J*= 8.0 Hz, H-6 of nicotinic hydrazide), 9.07 (1H, s, H-2 of nicotinic hydrazide), 13.79 (1H, s, NH); ^{13}C NMR δ *ppm*: 20.36 ($-CH(\underline{C}H_3)_2$), 27.22 ($-\underline{C}H(CH_3)_2$), 47.23 (N-CH_2), 112.59, 116.16, 119.04, 120.83, 124.59, 127.93, 131.68, 134.23, 136.06, 139.96, 142.62, 149.07, 154.42, 161.50 (C=O nicotinic hydrazide), 163.42 (C=O 2-indolinone); IR (KBr, ν cm^{-1}) 3450 (NH) and 1705, 1682 (2C=O); Analysis calculated for $C_{18}H_{17}ClN_4O_2$: C, 60.59; H, 4.80; N, 15.70; found C, 60.79; H, 4.75; N, 15.78.

N'-(5-Bromo-2-oxo-1-propylindolin-3-ylidene)nicotinohydrazide ***5g***.

Yellow powder, m.p. 167-168 °C; yield 73%; ^{1}H NMR δ *ppm*: 0.90 (3H, t, *J*= 8.0 Hz, $-CH_2C\underline{H_3}$), 1.67 (2H, brs, N-$CH_2C\underline{H_2}$), 3.75 (2H, brs, N-CH_2),7.25 (1H, d, *J*= 8.0 Hz, H-7 of 2-indolinone), 7.66-7.73 (3H, m, H-4 and H-6 of 2-indolinone, H-5 of nicotinic hydrazide), 8.28 (1H, brs, H-4 of nicotinic hydrazide), 8.87 (1H, brs, H-6 of nicotinic hydrazide), 9.08 (1H, s, H-2 of nicotinic hydrazide), 13.73 (1H, s, NH); ^{13}C NMR δ *ppm*: 11.61 ($-CH_2\underline{C}H_3$), 20.80 (N-$CH_2\underline{C}H_2$), 41.47 (N-CH_2), 112.87, 115.55, 121.61, 123.59, 124.58, 126.03, 128.31, 134.57, 137.48, 142.76, 149.02, 153.71, 161.21 (C=O nicotinic hydrazide), 163.83 (C=O 2-indolinone); IR (KBr, ν cm^{-1}) 3449 (NH) and 1694, 1673 (2C=O); Analysis calculated for $C_{17}H_{15}BrN_4O_2$: C, 52.73; H, 3.90; N, 14.47; found C, 52.57; H, 3.92; N, 14.51.

N'-(5-Bromo-1-isobutyl-2-oxindolin-3-ylidene)nicotinohydrazide ***5h***.

Orange powder, m.p. 174-175 °C; yield 75%; ^{1}H NMR δ *ppm*: 0.90 (6H, d, *J*= 6.8 Hz, $-CH(C\underline{H_3})_2$), 2.03-2.10 (1H, m, $-C\underline{H}(CH_3)_2$), 3.74 (2H, d, *J*= 8.0 Hz, N-CH_2), 7.21 (1H, d, *J*= 8.0 Hz, H-7 of 2-indolinone), 7.63-7.67 (2H, m, H-6 of of 2-indolinone and H-5 of nicotinic hydrazide), 7.75 (1H, s, H-4 of 2-indolinone),8.27 (1H, d, *J*= 8.0 Hz, H-4 of nicotinic hydrazide), 8.83 (1H, d, *J*= 7.6 Hz, H-6 of nicotinic hydrazide), 9.06 (1H, s, H-2 of nicotinic hydrazide), 13.79 (1H, s, NH); IR (KBr, ν cm^{-1}) 3449 (NH) and 1707, 1681 (2C=O); Analysis calculated for $C_{18}H_{17}BrN_4O_2$: C, 53.88; H, 4.27; N, 13.96; found C, 53.11; H, 4.23; N, 14.08.

Ethyl-2-(5-bromo-3-(2-nicotinoylhydrazono)-2-oxindolin-1-yl)acetate ***5i***.

Yellow powder, m.p. 206-208 °C; yield 81%; ^{1}H NMR δ *ppm*: 1.22 (3H, t, *J*= 8.0 Hz, $-CH_2C\underline{H_3}$), 4.18 (2H, q, *J*= 8.0 Hz, $-C\underline{H_2}CH_3$), 4.78 (2H, s, N-CH_2), 7.04 (1H, d, *J*= 8.4 Hz, H-5 of

nicotinic hydrazide), 7.21-7.24 (2H, m, H-5 and H-7 of 2-indolinone), 7.47 (1H, t, *J*= 8.0 Hz, H-6 of 2-indolinone), 7.68 (1H, d, *J*= 7.6 Hz, H-4 of 2-indolinone), 8.17 (1H, d, *J*= 8.8 Hz, H-4 of nicotinic hydrazide), 8.77 (1H, s, H-2 of nicotinic hydrazide), 13.52 (1H, s, NH); IR (KBr, *v* cm^{-1}) 3230 (NH) and 1741, 1711, 1687 (3C=O); Analysis calculated for $C_{18}H_{15}BrN_4O_4$: C, 50.13; H, 3.51; N, 12.99; found C, 50.35; H, 3.48; N, 13.06.

6-Methoxy-N'-(2-oxo-1-propylindolin-3-ylidene)nicotinohydrazide ***5j***.

Orange powder, m.p. 139-141 °C; yield 69%; ^{1}H NMR *δ ppm*: 0.91 (3H, t, *J*= 8.0 Hz, -$CH_2C\underline{H_3}$), 1.64-1.73 (2H, m, *N*-$CH_2C\underline{H_2}$), 3.74 (2H, t, *J*= 8.0 Hz, *N*-CH_2), 3.97 (3H, s, OCH_3), 7.04 (1H, d, *J*= 8.0 Hz, H-5 of nicotinic hydrazide), 7.17 (1H, t, *J*= 8.0 Hz, H-5 of 2-indolinone), 7.25 (1H, d, *J*= 8.0 Hz, H-7 of 2-indolinone), 7.47 (1H, t, *J*= 8.0 Hz, H-6 of 2-indolinone), 7.64 (1H, d, *J*= 8.0 Hz, H-4 of 2-indolinone), 8.16 (1H, d, *J*= 8.0 Hz, H-4 of nicotinic hydrazide), 8.76 (1H, s, H-2 of nicotinic hydrazide), 13.76 (1H, s, NH); ^{13}C NMR *δ ppm*: 11.65 (-$CH_2\underline{C}H_3$), 20.87 (*N*-$CH_2\underline{C}H_2$), 41.31 (*N*-CH_2), 54.46 (OCH_3), 110.74, 111.50, 119.57, 121.28, 122.09, 123.72, 128.01, 132.27, 138.90, 143.52, 148.41, 154.09, 161.62 (C=O nicotinic hydrazide), 163.18 (C=O 2-indolinone); IR (KBr, *v* cm^{-1}) 3449 (NH) and 1694, 1604 (2C=O); Analysis calculated for $C_{18}H_{18}N_4O_3$: C, 63.89; H, 5.36; N, 16.56; found C, 63.73; H, 5.42; N, 16.59.

N'-(1-Isobutyl-2-oxindolin-3-ylidene)-6-methoxynicotinohydrazide ***5k***.

Yellow powder, m.p. 154-156 °C; yield 74%; ^{1}H NMR *δ ppm*: 0.92 (6H, d, *J*= 6.8 Hz, -$CH(C\underline{H_3})_2$), 2.076-2.13 (1H, m, -$C\underline{H}(CH_3)_2$), 3.57 (2H, d, *J*= 7.2 Hz, *N*-CH_2), 3.96 (3H, s, OCH_3), 7.01 (1H, d, *J*= 8.4 Hz, H-5 of nicotinic hydrazide), 7.15 (1H, t, *J*= 7.6 Hz, H-5 of 2-indolinone), 7.22 (1H, d, *J*= 8.0 Hz, H-7 of 2-indolinone), 7.44 (1H, t, *J*= 8.0 Hz, H-6 of 2-indolinone), 7.62 (1H, d, *J*= 7.6 Hz, H-4 of 2-indolinone), 8.13 (1H, d, *J*= 8.8 Hz, H-4 of nicotinic hydrazide), 8.74 (1H, s, H-2 of nicotinic hydrazide), 13.75 (1H, s, NH); ^{13}C NMR *δ ppm*: 20.45 (-$CH(\underline{C}H_3)_2$), 27.28 (-$\underline{C}H(CH_3)_2$), 47.10 (*N*-CH_2), 54.46 (OCH_3), 110.99, 111.52, 115.38, 119.51, 121.22, 122.07, 123.72, 132.22, 137.33, 138.86, 143.83, 148.36, 153.37, 161.84 (C=O nicotinic hydrazide), 166.50 (C=O 2-indolinone); IR (KBr, *v* cm^{-1}) 3450 (NH) and 1704, 1610 (2C=O); Analysis calculated for $C_{19}H_{20}N_4O_3$: C, 64.76; H, 5.72; N, 15.90; found C, 64.98; H, 5.68; N, 15.92.

Ethyl-2-(3-(2-(6-methoxynicotinoyl)hydrazono)-2-oxindolin-1-yl)acetate ***5l***.

Yellow powder, m.p. 132-133 °C; yield 70%; ^{1}H NMR δ *ppm*: 1.22 (3H, t, *J*= 8.0 Hz, -$CH_2C\underline{H_3}$), 3.97 (3H, s, OCH_3), 4.17 (2H, q, *J*= 8.0 Hz, -$C\underline{H_2}CH_3$), 4.74 (2H, s, *N*-CH_2), 7.02 (1H, d, *J*= 8.4 Hz, H-5 of nicotinic hydrazide), 7.20-7.23 (2H, m, H-5 and H-7 of 2-indolinone), 7.47 (1H, t, *J*= 8.0 Hz, H-6 of 2-indolinone), 7.67 (1H, d, *J*= 7.6 Hz, H-4 of 2-indolinone), 8.16 (1H, d, *J*= 8.8 Hz, H-4 of nicotinic hydrazide), 8.76 (1H, s, H-2 of nicotinic hydrazide), 13.51 (1H, s, NH); ^{13}C NMR δ *ppm*: 14.47 (-$CH_2\underline{C}H_3$), 41.40 (-$\underline{C}H_2CH_3$), 54.44 (OCH_3), 61.92 (*N*-CH_2), 110.84, 111.44, 119.39, 121.27, 121.98, 124.11, 132.25, 136.72, 139.00, 143.14, 148.35, 161.49 (C=O nicotinic hydrazide), 166.52 (C=O 2-indolinone), 167.86 (C=O ester); IR (KBr, ν cm^{-1}) 3451 (NH) and 1749, 1684, 1670 (3C=O); Analysis calculated for $C_{19}H_{18}N_4O_5$: C, 59.68; H, 4.75; N, 14.65; found C, 59.84; H, 4.71; N, 14.69.

N'-(1-Benzyl-2-oxindolin-3-ylidene)-6-methoxynicotinohydrazide ***5m***.

Yellow powder, m.p. 160-162 °C; yield 83%; ^{1}H NMR δ *ppm*: 3.96 (3H, s, OCH_3), 5.02 (2H, s, benzylic protons),7.01 (1H, d, *J*= 8.8 Hz, H-5 of nicotinic hydrazide), 7.07 (1H, d, *J*= 7.6 Hz, H-7 of 2-indolinone), 7.13 (1H, t, *J*= 8.0 Hz, H-5 of 2-indolinone), 7.27 (1H, t, *J*= 8.4 Hz, H-6 of 2-indolinone), 7.33 (2H, t, *J*= 7.6 Hz, H-3 and H-5 of benzyl moiety), 7.40-7.43 (3H, m, H-2, H-4 and H-6 of benzyl moiety),7.63 (1H, d, *J*= 7.6 Hz, H-4 of 2-indolinone), 8.16 (1H, d, *J*= 8.8 Hz, H-4 of nicotinic hydrazide), 8.77 (1H, s, H-2 of nicotinic hydrazide), 13.71 (1H, s, NH); ^{13}C NMR δ *ppm*: 43.10 (benzylic carbon), 54.43 (OCH_3), 111.01, 111.47, 119.71, 121.29, 122.04, 123.91, 127.94, 128.15, 128.96, 129.20, 132.12, 136.05, 137.29, 138.91, 143.13, 148.31, 161.60 (C=O nicotinic hydrazide), 166.51 (C=O 2-indolinone); IR (KBr, ν cm^{-1}) 3449 (NH) and 1694, 1602 (2C=O); Analysis calculated for $C_{22}H_{18}N_4O_3$: C, 68.38; H, 4.70; N, 14.50; found C, 68.17; H, 4.75; N, 14.44.

2-Methyl-N'-(2-oxo-1-propylindolin-3-ylidene)-6-phenylnicotinohydrazide ***9a***.

Orange powder, m.p. 91-93 °C; yield 76%; ^{1}H NMR δ *ppm*: 0.89 (3H, t, *J*= 8.0 Hz, -$CH_2C\underline{H_3}$), 1.62-1.71 (2H, m, *N*-$CH_2C\underline{H_2}$), 2.71 (3H, s, CH_3), 3.70 (2H, t, *J*= 8.0 Hz, *N*-CH_2), 7.15-7.23 (2H, m, H-5 and H-6 of 2-indolinone), 7.45-7.56 (5H, m, ArH of phenyl ring), 7.97 (1H, d, *J*= 8.0 Hz, H-7 of 2-indolinone), 8.07 (1H, d, *J*= 8.0 Hz, H-4 of 2-indolinone), 8.16 (2H, d, *J*= 8.0 Hz, H-4 and H-5 of nicotinic hydrazide), 13.31 (1H, s, NH); ; ^{13}C NMR δ *ppm*: 11.63 (-$CH_2C\underline{H_3}$), 20.85 (*N*-$CH_2C\underline{H_2}$), 23.88 (CH_3), 41.28 (*N*-CH_2), 110.68, 117.82, 119.51, 121.16, 123.65, 127.38, 129.30, 130.21, 132.29, 137.50, 138.17, 143.60, 144.83, 147.01, 151.56, 157.40,

161.33 (C=O nicotinic hydrazide), 163.10 (C=O 2-indolinone); IR (KBr, ν cm^{-1}) 3449 (NH) and 1701, 1693 (2C=O); Analysis calculated for $C_{24}H_{22}N_4O_2$: C, 72.34; H, 5.57; N, 14.06; found C, 72.39; H, 5.51; N, 14.02.

N'-(1-Isobutyl-2-oxindolin-3-ylidene)-2-methyl-6-phenylnicotinohydrazide ***9b***.

Yellow powder, m.p. 139-141 °C; yield 80%; ^{1}H NMR *δ ppm*: 0.91 (6H, d, *J*= 6.8 Hz, -CH(C$\underline{H_3}$)$_2$), 2.05-2.12 (1H, m, -C$\underline{H}$(CH$_3$)$_2$), 2.71 (3H, s, CH$_3$), 3.56 (2H, d, *J*= 8.0 Hz, *N*-CH$_2$), 7.16-7.24 (2H, m, H-5 and H-6 of 2-indolinone), 7.44-7.56 (5H, m, ArH of phenyl ring), 7.98 (1H, d, *J*= 8.0 Hz, H-7 of 2-indolinone), 8.07 (1H, d, *J*= 8.0 Hz, H-4 of 2-indolinone), 8.17 (2H, d, *J*= 8.0 Hz, H-4 and H-5 of nicotinic hydrazide), 13.42 (1H, s, NH); ^{13}C NMR *δ ppm*: 20.41 (-CH($\underline{C}$H$_3$)$_2$), 23.88 (-$\underline{C}$H(CH$_3$)$_2$), 27.24 (CH$_3$), 56.51 (*N*-CH$_2$), 110.91, 117.79, 119.45, 121.13, 123.66, 127.38, 129.30, 130.21, 132.26, 137.36, 138.17, 143.91, 144.93, 147.07, 151.66, 157.47, 161.54 (C=O nicotinic hydrazide), 163.10 (C=O 2-indolinone); IR (KBr, ν cm^{-1}) 3401 (NH) and 1690, 1683 (2C=O); Analysis calculated for $C_{25}H_{24}N_4O_2$: C, 72.80; H, 5.86; N, 13.58; found C, 72.86; H, 5.81; N, 13.53.

Ethyl-2-(3-(2-(2-methyl-6-phenylnicotinoyl)hydrazono)-2-oxindolin-1-yl)acetate ***9c***.

Yellow powder, m.p. 156-157 °C; yield 73%; ^{1}H NMR *δ ppm*: 1.21 (3H, t, *J*= 8.0 Hz, -CH$_2$C$\underline{H_3}$), 2.71 (3H, s, CH$_3$), 4.16 (2H, q, *J*= 8.0 Hz, -C$\underline{H_2}$CH$_3$), 4.72 (2H, s, *N*-CH$_2$), 7.17-7.22 (2H, m, H-5 and H-6 of 2-indolinone), 7.46-7.57 (5H, m, ArH of phenyl ring), 7.98 (1H, d, *J*= 8.0 Hz, H-7 of 2-indolinone), 8.09 (1H, d, *J*= 8.0 Hz, H-4 of 2-indolinone), 8.17 (2H, d, *J*= 8.0 Hz, H-4 and H-5 of nicotinic hydrazide), 13.14 (1H, s, NH); ^{13}C NMR *δ ppm*: 14.47 (-CH$_2$$\underline{C}H_3$), 23.88 (CH$_3$), 41.38 (-$\underline{C}H_2CH_3$), 61.92 (*N*-CH$_2$), 110.82, 112.38, 117.70, 119.36, 121.17, 124.10, 127.40, 129.33, 130.25, 132.32, 134.07, 138.16, 143.24, 144.93, 147.07, 151.66, 157.50, 161.20 (C=O nicotinic hydrazide), 163.19 (C=O 2-indolinone), 167.89 (C=O ester); IR (KBr, ν cm^{-1}) 3449 (NH) and 1737, 1691, 1616 (3C=O); Analysis calculated for $C_{25}H_{22}N_4O_4$: C, 67.86; H, 5.01; N, 12.66; found C, 67.82; H, 5.06; N, 12.61.

5-Bromo-N-(6-methoxypyridin-3-yl)-2,3-dioxindoline-1-carboxamide ***14***.

Yellow powder, m.p. 282-284 °C; yield 81%; ^{1}H NMR *δ ppm*: 3.82 (3H, s, OCH$_3$), 6.77 (1H, d, *J*= 8.0 Hz, H-7 of indoline-2,3-dione), 6.88 (1H, d, *J*= 8.0 Hz, H-6 of indoline-2,3-dione), 7.66 (1H, s, H-4 of of indoline-2,3-dione), 7.73 (1H, d, *J*= 8.0 Hz, H-5 of nicotinic hydrazide), 7.81

(1H, d, *J*= 8.0 Hz, H-4 of nicotinic hydrazide), 8.19 (1H, s, H-2 of nicotinic hydrazide), 8.62 (1H, s, NH); ^{13}C NMR δ *ppm*: 53.53 (OCH_3), 110.44, 112.67, 120.01, 125.15, 127.36, 130.90, 131.89, 137.63, 138.85, 140.52, 150.03, 153.69 (C=O amid), 159.57 (C=O of position 2 of indoline-2,3-dione), 183.65 (C=O of position 3 of indoline-2,3-dione); IR (KBr, ν cm^{-1}) 3279 (NH) and 1730, 1697, 1687 (3C=O); Analysis calculated for $C_{15}H_{10}BrN_3O_4$: C, 47.90; H, 2.68; N, 11.17; found C, 48.03; H, 2.69; N, 11.22.

2. Microplate Alamar Blue Anti-Tubercular Assay

Microplate alamar blue assay (MABA) was used to determine MICs of the prepared hybrids (**5a-m**, **9a-c** and **14**) against M. tuberculosis ATCC 27294 (Isoniazid-sensitive strain) and Mycobacterium tuberculosis ATCC 35823 (resistant to Isoniazid and Streptomycin). Isoniazid was used as a reference drug against sensitive strain. Preparation of the inoculum was done using fresh Lowenstein Jensen (LJ) medium re-suspended in 7H9-S medium (7H9 broth, 0.1% casitone, 0.5% glycerol, supplemented oleic acid, albumin, dextrose, and catalase (OADC), adjusted to a McFarland tube No. 1, and diluted 1:20; 100 µl was used as inoculum. The examined hybrids were dissolved in DMSO. Drug-free controls containing broth with DMSO were included in the experiment. The final concentration of DMSO in the test medium did not exceed 0.5% (v/v) of the total solution composition, which had no effect on the growth of *M. tuberculosis*. The 96 wells plates were treated with 100 µl of two-fold serial dilution of each compound. Final concentrations of the examined hybrids in wells were 1000- 0.006 µg/mL. Sterile deionized water (200 µl) was added to all outer-perimeter wells of sterile 96 well plates to decrease evaporation of the medium in the test wells during incubation. A growth control without antibiotic and a sterile control were also set on each plate. The plate was then covered, sealed in plastic bags and incubated at 37 °C in normal atmosphere. After 7 days of incubation, each well was supplied with 30 µl of the Alamar blue solution, and then the plate was re-incubated overnight. Colour change from blue (oxidized state) to pink (reduced) highlighted the growth of bacteria. The MIC was expressed as the minimum concentration of compound which prohibited blue to pink colour change. MIC values were calculated in µg/mL.

3. XTT Susceptibility Antibacterial Assay

Microorganisms: Gram negative bacteria: *Mycoplasma pneumoniae ATCC 15531*, *Haemophilus influenzae ATCC 10211*, *Moraxella catarrhalis ATCC 25238*, *Klebsiella pneumoniae ATCC 43816* and *Bordetella pertussis ATCC 9340*, in addition to *Streptococcus pneumoniae ATCC 1659*, representing Gram positive bacterium, All strains are American type culture collection (ATCC).

Colorimetric broth micro-dilution method using XTT [2,3-bis(2-methoxy-4-nitro-5-sulfo-phenyl)-2H-tetrazolium-5-carboxanilide]-reduction assay (33, 34) was adopted to determine the minimum inhibitory concentration (MIC) of examined hybrids against bacteria causing bronchitis. All bacterial strains were cultured overnight at 37 °C in Tryptone Soya Broth (TSB) (Oxoid, UK). XTT (Sigma) was prepared in a saturated solution at 0.5 g/L in Ringer's lactate. The solution was sterilized through a 0.22-µm-pore-size filter. The compounds were serially diluted in DMSO, and then 50 µL of each dilution at final concentrations of (1000- 0.24 µg/ mL) were added to wells in Microtiter plate (96 wells) containing 100 µL TSB. Fifty µL of adjusted microbial inoculum (106 CFU/mL) was added to each well, and then the Microtiter plates were incubated in the dark at 37 °C for 24 h. After incubation, 100 µL of freshly prepared XTT were added, incubated again for 1 h at 37 °C. Colorimetric variation in the XTT assay was measured using a Microtiter plate reader (BioTECK, USA) at 492 NM. The MIC was specified as the extract concentration that produced a 100 % decrease in optical density compared with control growth results. Azithromycin was used as a standard antibacterial.

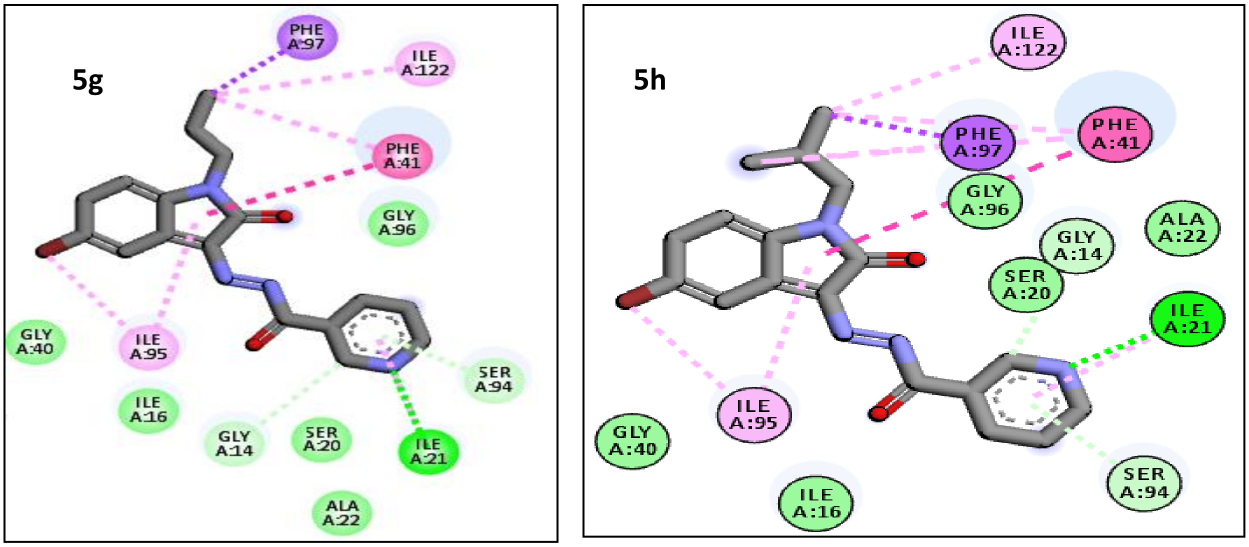

Fig. S1. The 2D diagrams for interactions of compounds **5g** and **5h** in InhA active site.

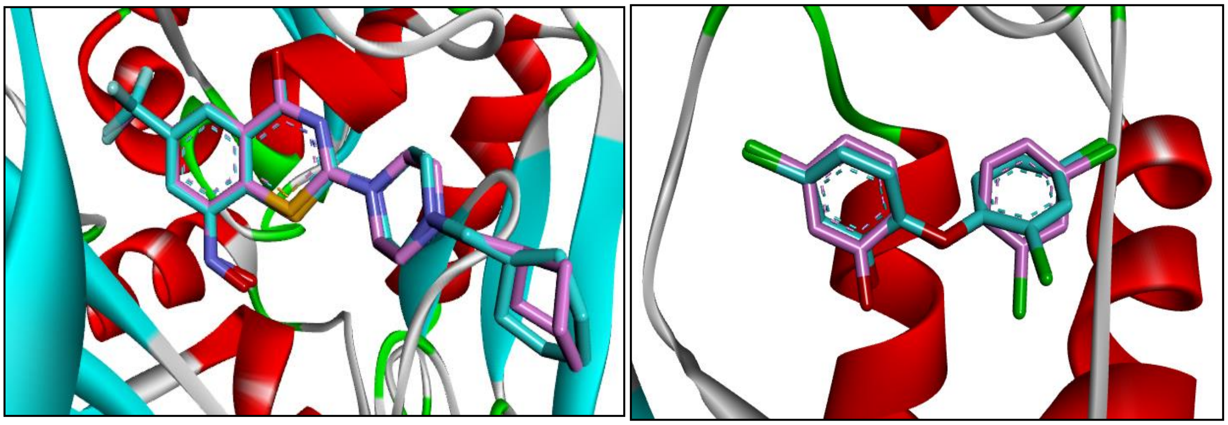

DprE1 **InhA**

Fig. S2. the 3D illustrations of the superimposition for the docking poses and the co-crystalized ligands in DprE1 and InhA active sites.

Table S3. Summarized interactions between compounds **5g** and **5h** with the DprE1 active site

Compound	Bond	Distance (A)
**5g**	Hydrogen bond with Asparagine 385	2.66
	Hydrogen bond with Asparagine 385	2.77
	Hydrogen bond with Histidine 132	2.68
	Hydrogen bond with Histidine 132	3.04
	Hydrogen bond with Tyrosine 415	2.09
	Non-classical Hydrogen bond with Glycine 133	2.42
	Non-classical Hydrogen bond with Proline 116	2.95
	Pi-Pi interaction with Histidine 132	5.44
	Pi-Alkyl interaction with Proline 116	5.17
	Pi-Alkyl interaction with Valine 365	4.76
	Pi-Alkyl interaction with Lysine 367	4.33
	Alkyl-Alkyl interaction with Leucine 131	3.75
**5h**	Hydrogen bond with Asparagine 385	2.75
	Hydrogen bond with Cysteine 387	3.72
	Hydrogen bond with Histidine 132	2.68
	Hydrogen bond with Histidine 132	3.04
	Hydrogen bond with Tyrosine 415	2.09
	Non-classical Hydrogen bond with Glycine 133	2.42
	Non-classical Hydrogen bond with Proline 116	2.95
	Pi-Pi interaction with Histidine 132	5.44
	Pi-Alkyl interaction with Proline 116	5.17
	Pi-Alkyl interaction with Valine 365	4.75
	Pi-Alkyl interaction with Lysine 367	4.39
	Alkyl-Alkyl interaction with Leucine 131	4.66
	Alkyl-Alkyl interaction with Valine 121	4.49
	Alkyl-Alkyl interaction with Alanine 417	3.87
	Alkyl-Alkyl interaction with Proline 116	4.45

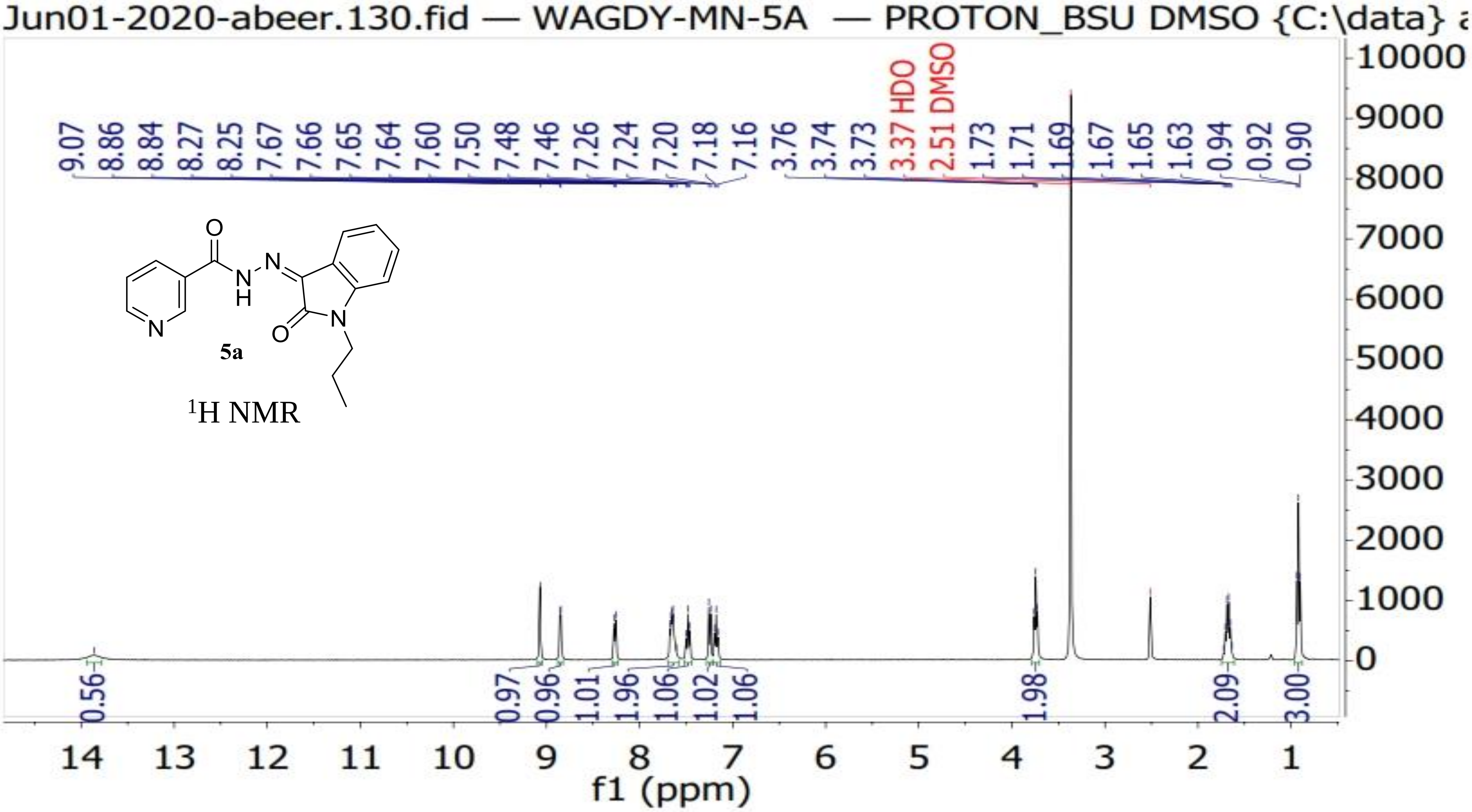
Jun01-2020-abeer.130.fid — WAGDY-MN-5A — PROTON_BSU DMSO {C:\data} a
9.07 8.86 8.84 8.27 8.25 7.67 7.66 7.65 7.64 7.60 7.50 7.48 7.46 7.26 7.24 7.20 7.18 7.16 3.76 3.74 3.73 3.37 HDO 2.51 DMSO 1.73 1.71 1.69 1.67 1.65 1.63 0.94 0.92 0.90
5a
¹H NMR
0.56 0.97 0.96 1.01 1.96 1.06 1.02 1.06 1.98 2.09 3.00
f1 (ppm)

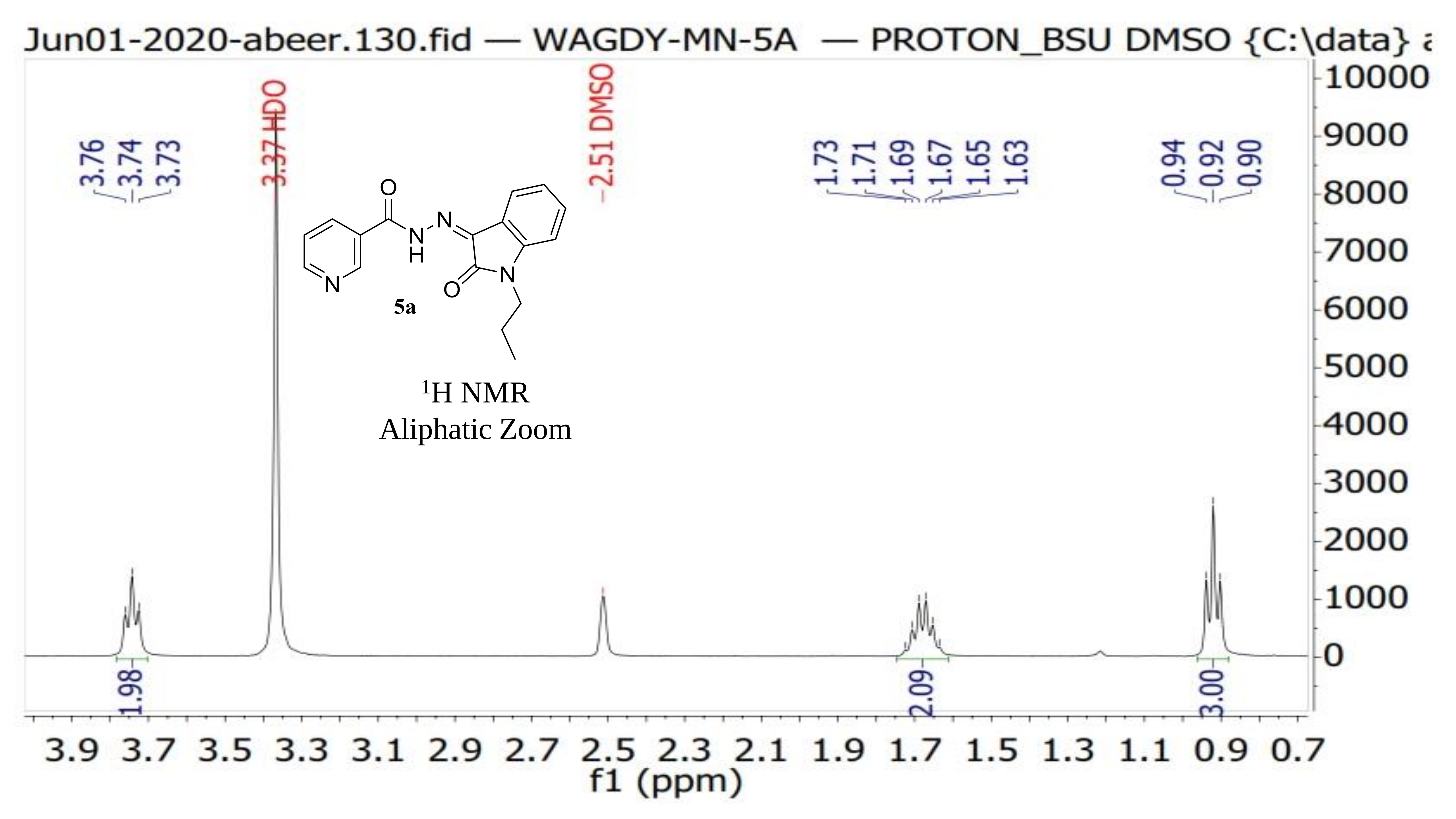
Jun01-2020-abeer.130.fid — WAGDY-MN-5A — PROTON_BSU DMSO {C:\data} a
3.76
3.74
3.73
3.37 HDO
2.51 DMSO
1.73
1.71
1.69
1.67
1.65
1.63
0.94
0.92
0.90
O
N
H
N
N
O
5a
1H NMR
Aliphatic Zoom
1.98
2.09
3.00
10000
9000
8000
7000
6000
5000
4000
3000
2000
1000
0
3.9 3.7 3.5 3.3 3.1 2.9 2.7 2.5 2.3 2.1 1.9 1.7 1.5 1.3 1.1 0.9 0.7
f1 (ppm)

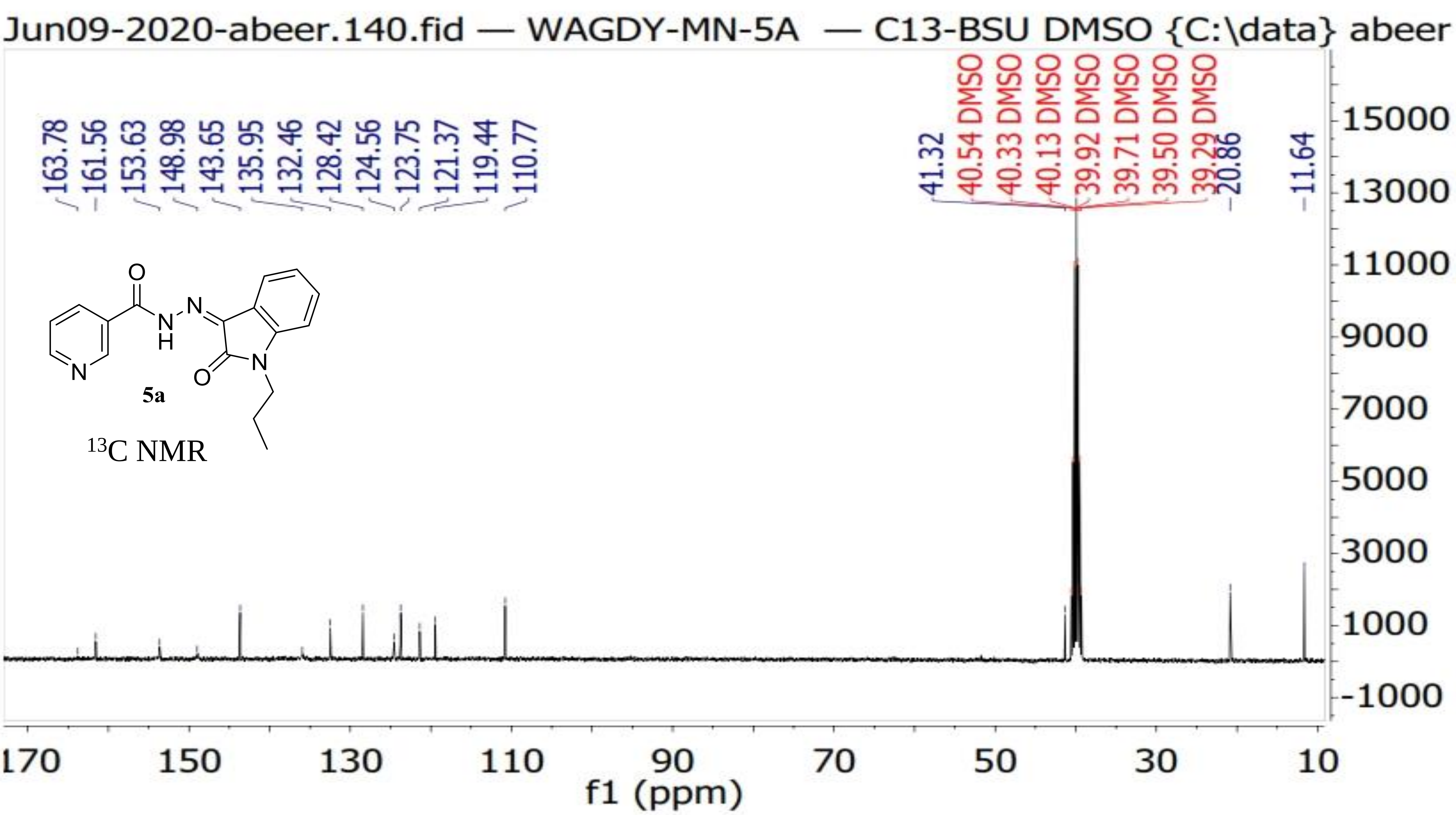
Jun09-2020-abeer.140.fid — WAGDY-MN-5A — C13-BSU DMSO {C:\data} abeer
163.78
161.56
153.63
148.98
143.65
135.95
132.46
128.42
124.56
123.75
121.37
119.44
110.77
41.32
40.54 DMSO
40.33 DMSO
40.13 DMSO
39.92 DMSO
39.71 DMSO
39.50 DMSO
39.29 DMSO
20.86
11.64
5a
13C NMR
f1 (ppm)

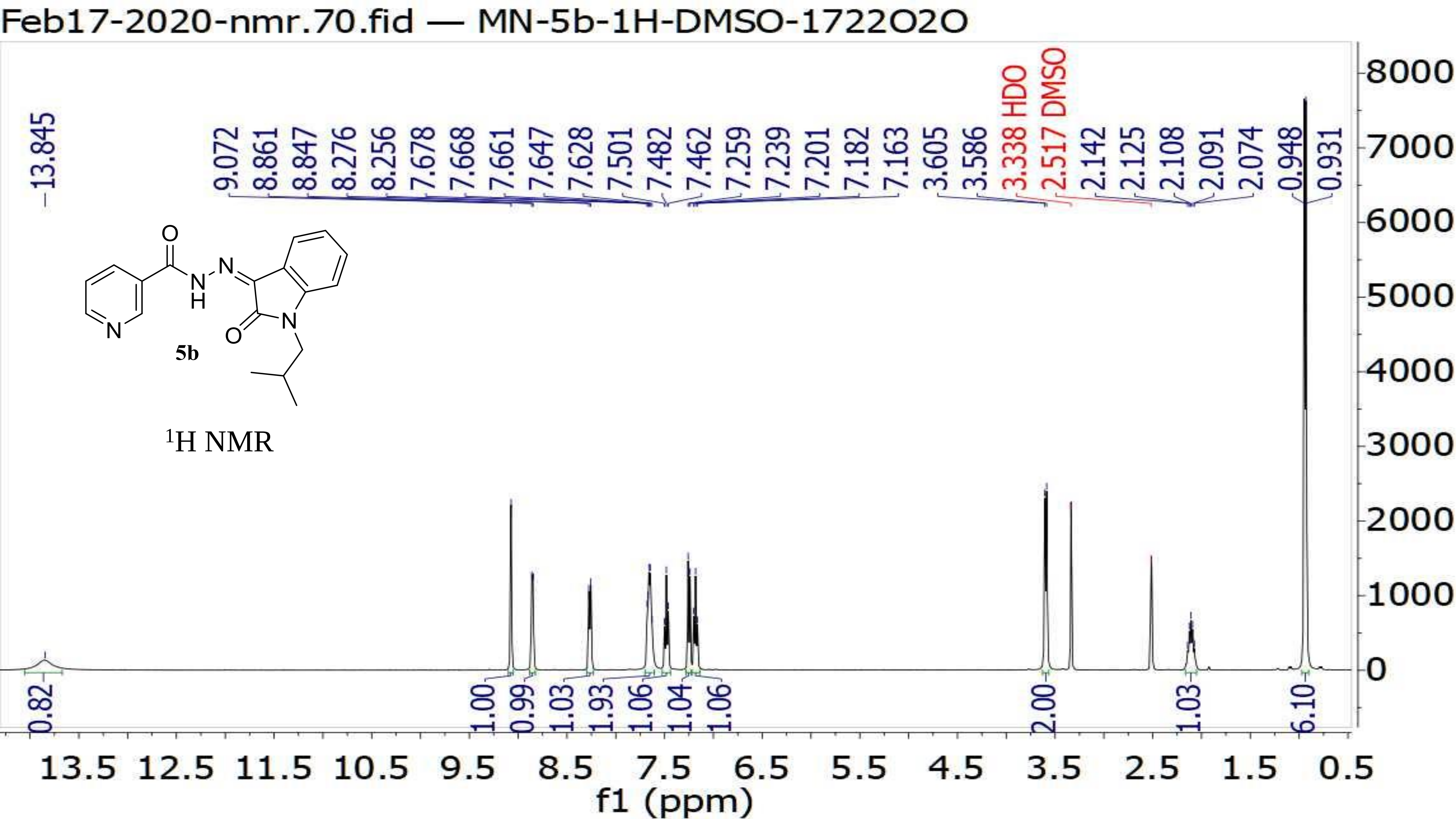
Feb17-2020-nmr.70.fid — MN-5b-1H-DMSO-172202O
-13.845
9.072
8.861
8.847
8.276
8.256
7.678
7.668
7.661
7.647
7.628
7.501
7.482
7.462
7.259
7.239
7.201
7.182
7.163
3.605
3.586
3.338 HDO
2.517 DMSO
2.142
2.125
2.108
2.091
2.074
0.948
0.931
5b
1H NMR
0.82
1.00
0.99
1.03
1.93
1.06
1.04
1.06
2.00
1.03
6.10
13.5
12.5
11.5
10.5
9.5
8.5
7.5
6.5
5.5
4.5
3.5
2.5
1.5
0.5
f1 (ppm)
8000
7000
6000
5000
4000
3000
2000
1000
0

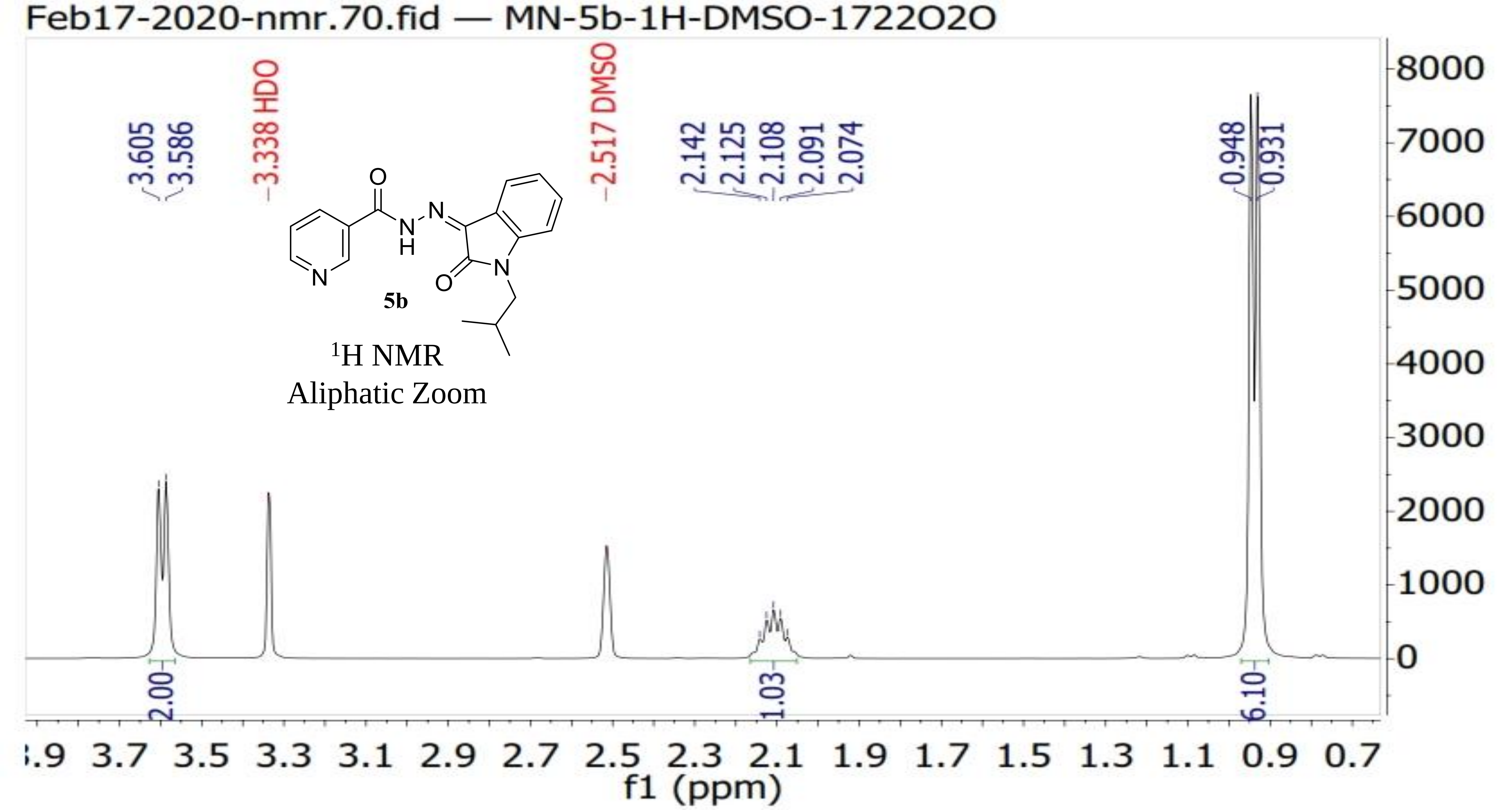
Feb17-2020-nmr.70.fid — MN-5b-1H-DMSO-1722O2O
3.605
3.586
3.338 HDO
2.517 DMSO
2.142
2.125
2.108
2.091
2.074
0.948
0.931
5b
1H NMR
Aliphatic Zoom
2.00
1.03
6.10
f1 (ppm)

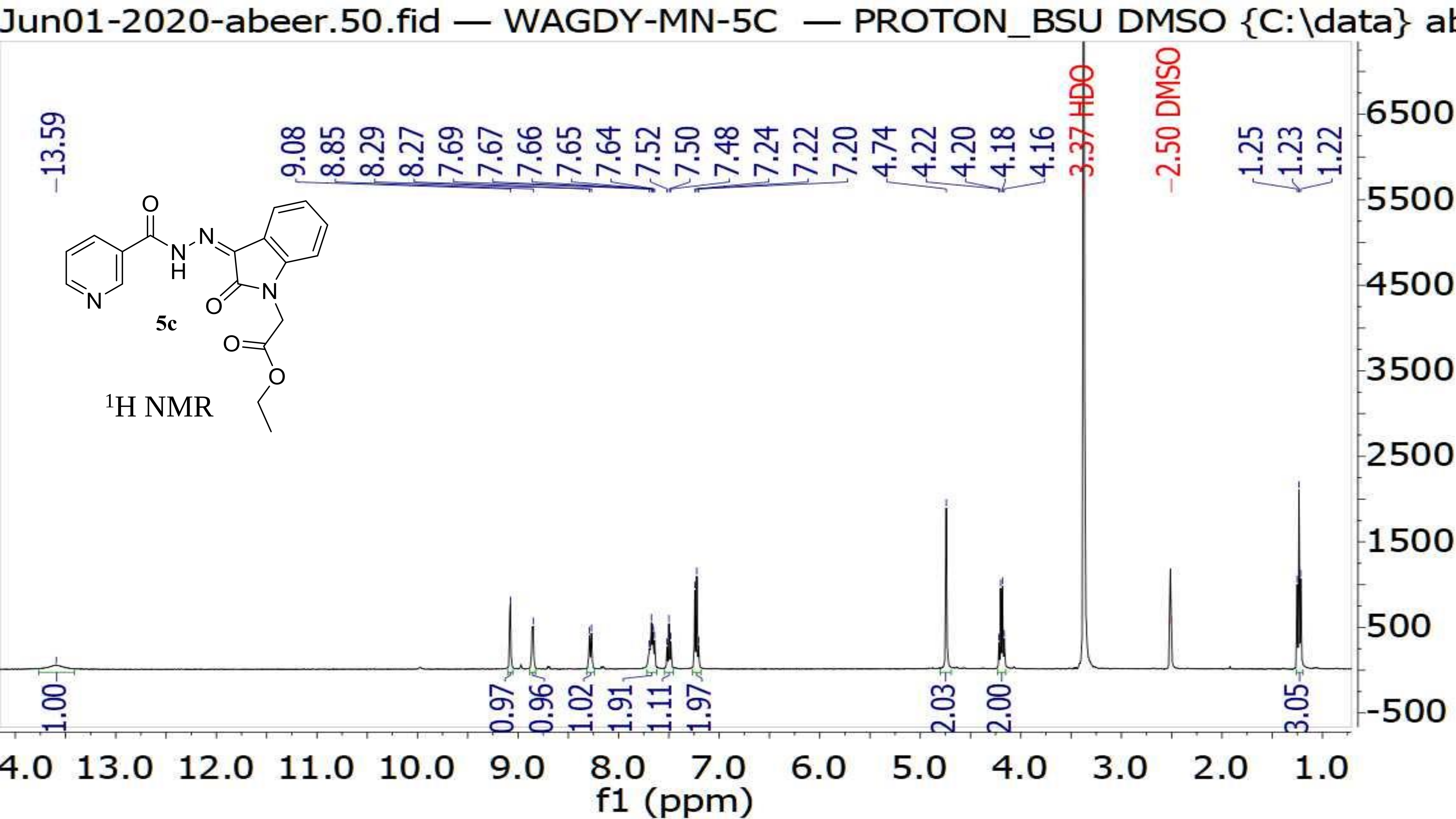
Jun01-2020-abeer.50.fid — WAGDY-MN-5C — PROTON_BSU DMSO {C:\data} ab
13.59
9.08 8.85 8.29 8.27 7.69 7.67 7.66 7.65 7.64 7.52 7.50 7.48 7.24 7.22 7.20 4.74 4.22 4.20 4.18 4.16
3.37 HDO
2.50 DMSO
1.25 1.23 1.22
5c
1H NMR
1.00
0.97 0.96 1.02 1.91 1.11 1.97
2.03 2.00
3.05
6500
5500
4500
3500
2500
1500
500
-500
4.0 13.0 12.0 11.0 10.0 9.0 8.0 7.0 6.0 5.0 4.0 3.0 2.0 1.0
f1 (ppm)

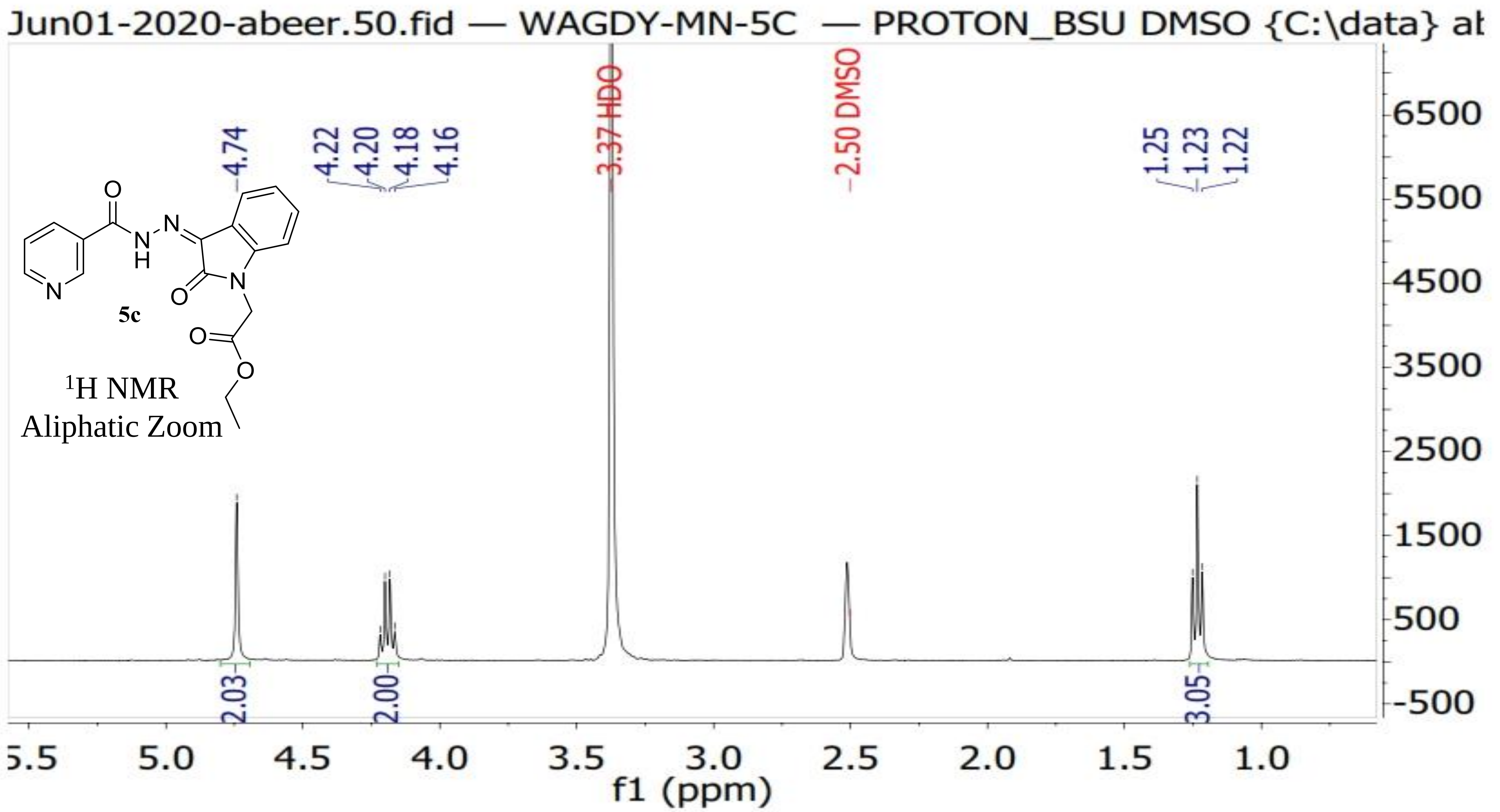
Jun01-2020-abeer.50.fid — WAGDY-MN-5C — PROTON_BSU DMSO {C:\data} at
4.74
4.22
4.20
4.18
4.16
3.37 HDO
2.50 DMSO
1.25
1.23
1.22
5c
1H NMR
Aliphatic Zoom
2.03
2.00
3.05
6500
5500
4500
3500
2500
1500
500
-500
5.5
5.0
4.5
4.0
3.5
3.0
2.5
2.0
1.5
1.0
f1 (ppm)

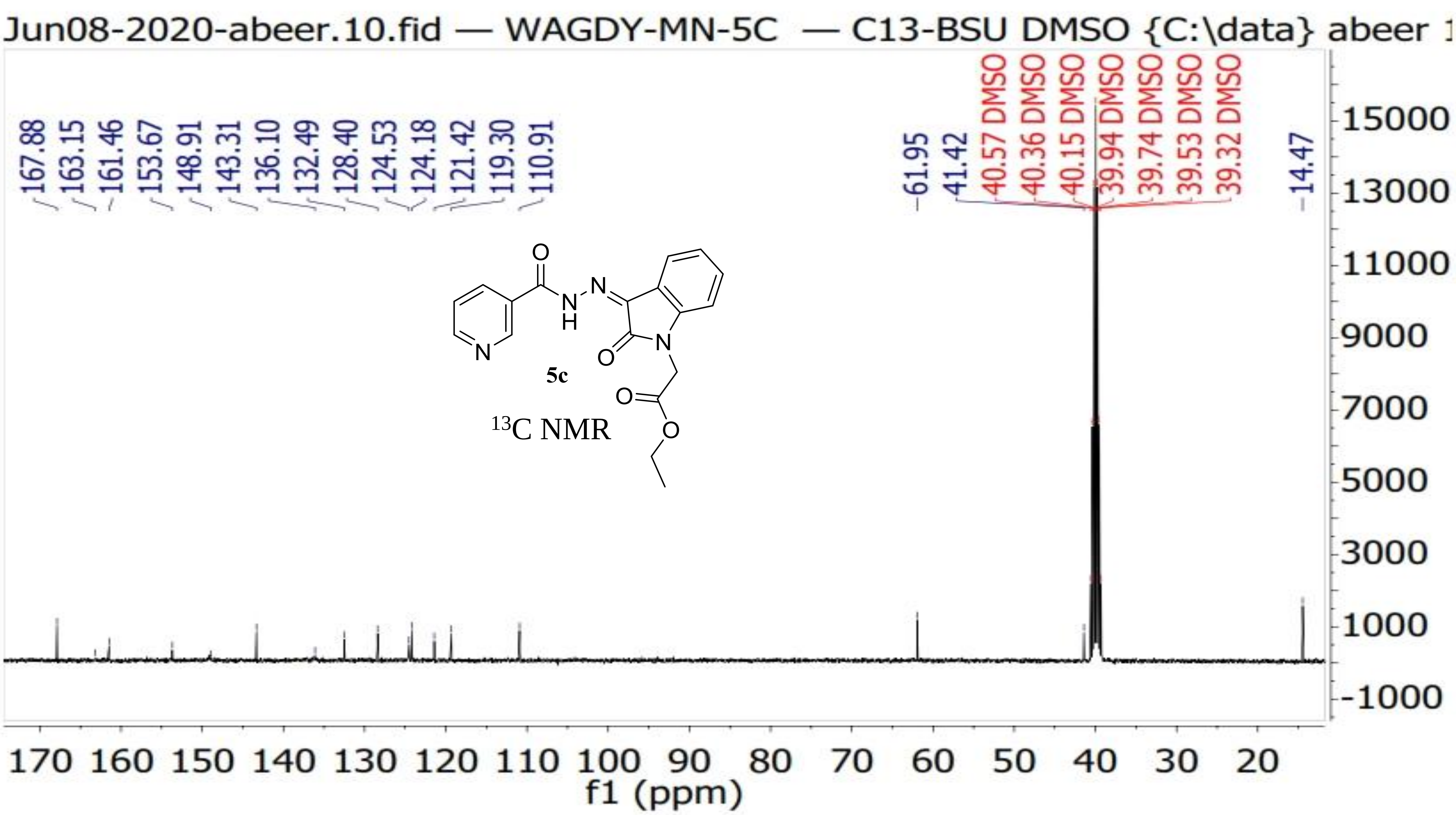
Jun08-2020-abeer.10.fid — WAGDY-MN-5C — C13-BSU DMSO {C:\data} abeer 1
167.88
163.15
161.46
153.67
148.91
143.31
136.10
132.49
128.40
124.53
124.18
121.42
119.30
110.91
61.95
41.42
40.57 DMSO
40.36 DMSO
40.15 DMSO
39.94 DMSO
39.74 DMSO
39.53 DMSO
39.32 DMSO
14.47
5c
13C NMR
f1 (ppm)

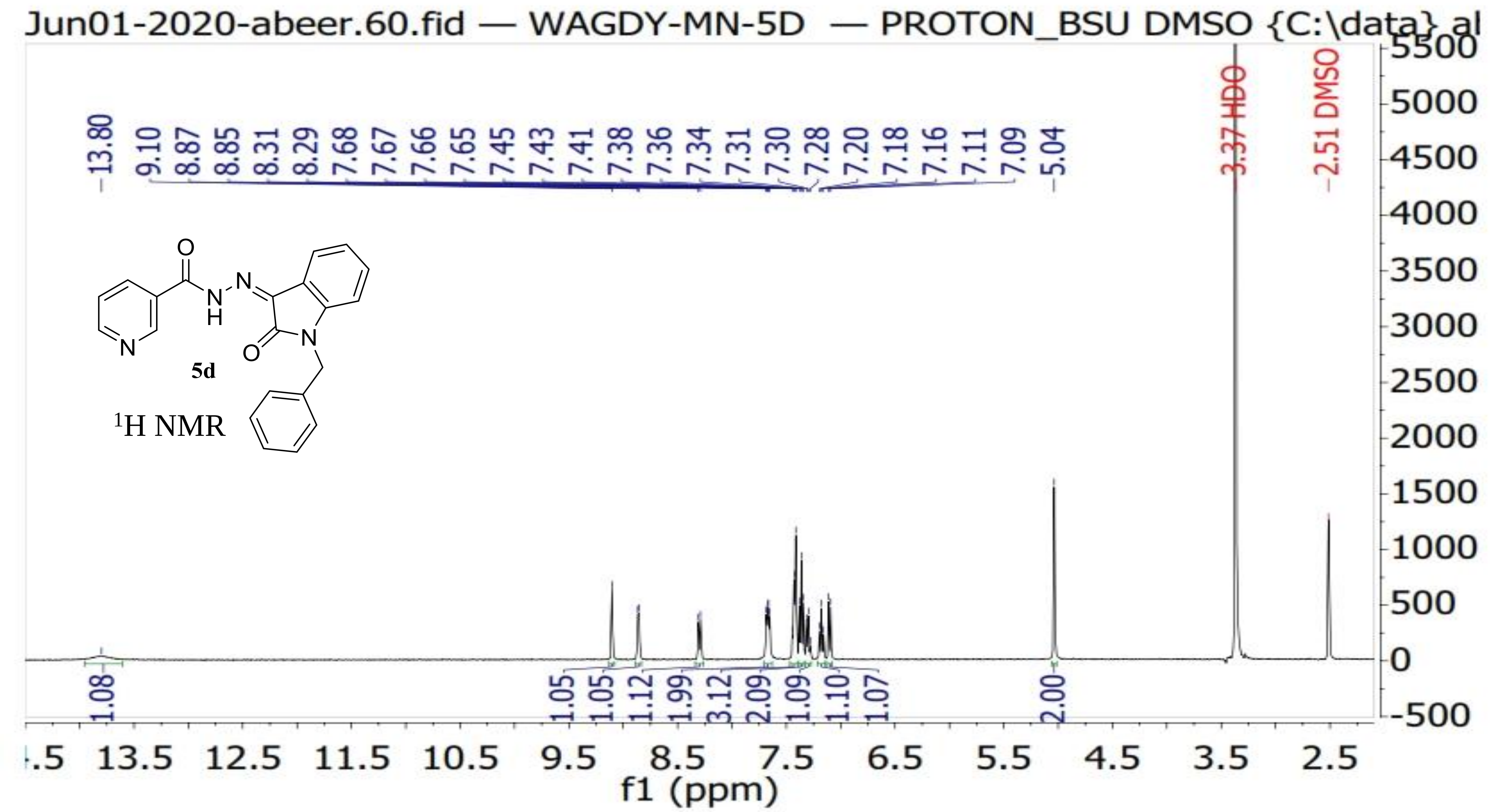

Jun01-2020-abeer.60.fid — WAGDY-MN-5D — PROTON_BSU DMSO {C:\data} al
13.80
9.10 8.87 8.85 8.31 8.29 7.68 7.67 7.66 7.65 7.45 7.43 7.41 7.38 7.36 7.34 7.31 7.30 7.28 7.20 7.18 7.16 7.11 7.09
5.04
3.37 HDO
2.51 DMSO
5d
1H NMR
1.08
1.05 1.05 1.12 1.99 3.12 2.09 1.09 1.10 1.07
2.00
f1 (ppm)

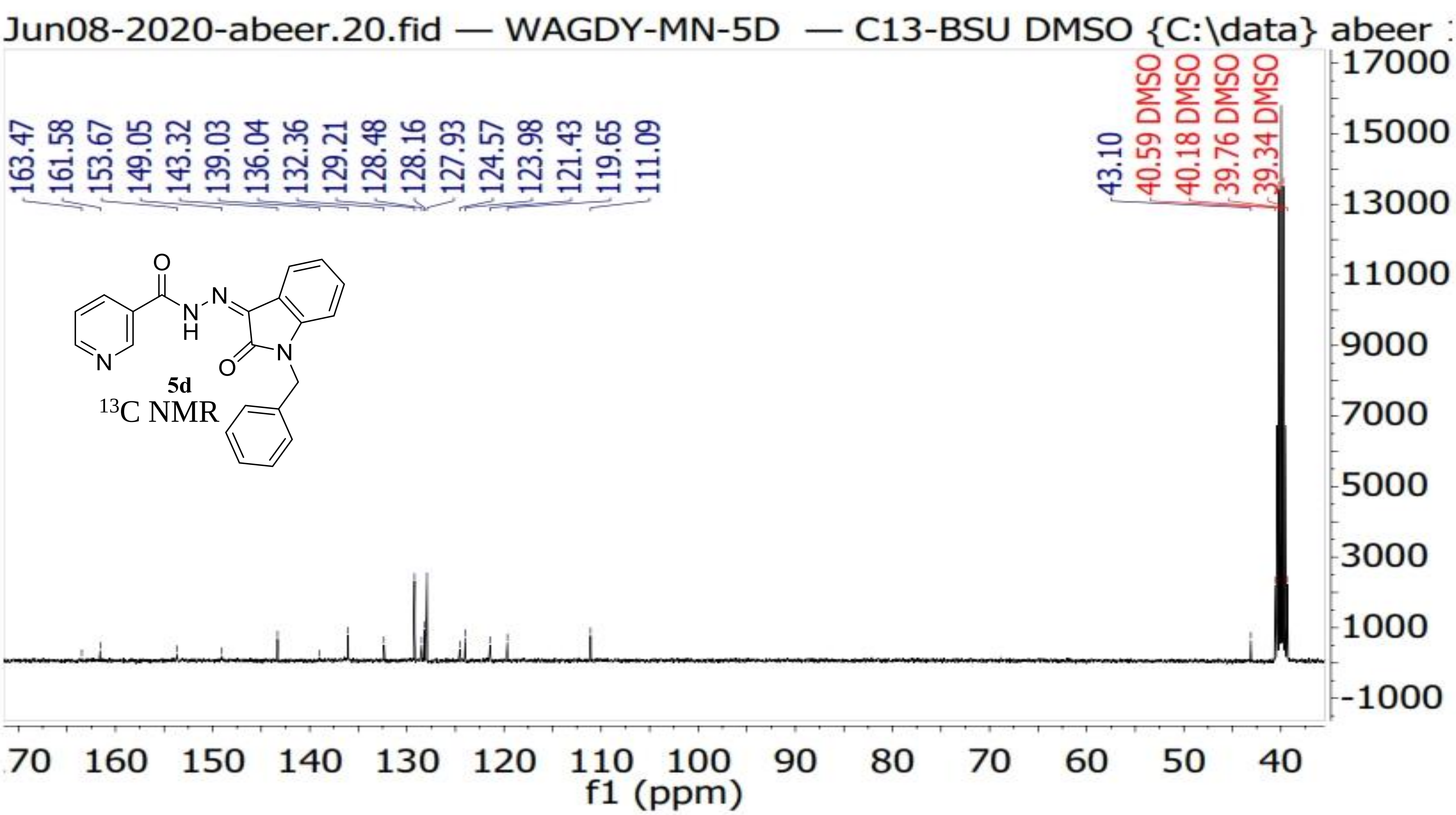
Jun08-2020-abeer.20.fid — WAGDY-MN-5D — C13-BSU DMSO {C:\data} abeer
163.47
161.58
153.67
149.05
143.32
139.03
136.04
132.36
129.21
128.48
128.16
127.93
124.57
123.98
121.43
119.65
111.09
43.10
40.59 DMSO
40.18 DMSO
39.76 DMSO
39.34 DMSO
5d
13C NMR
f1 (ppm)

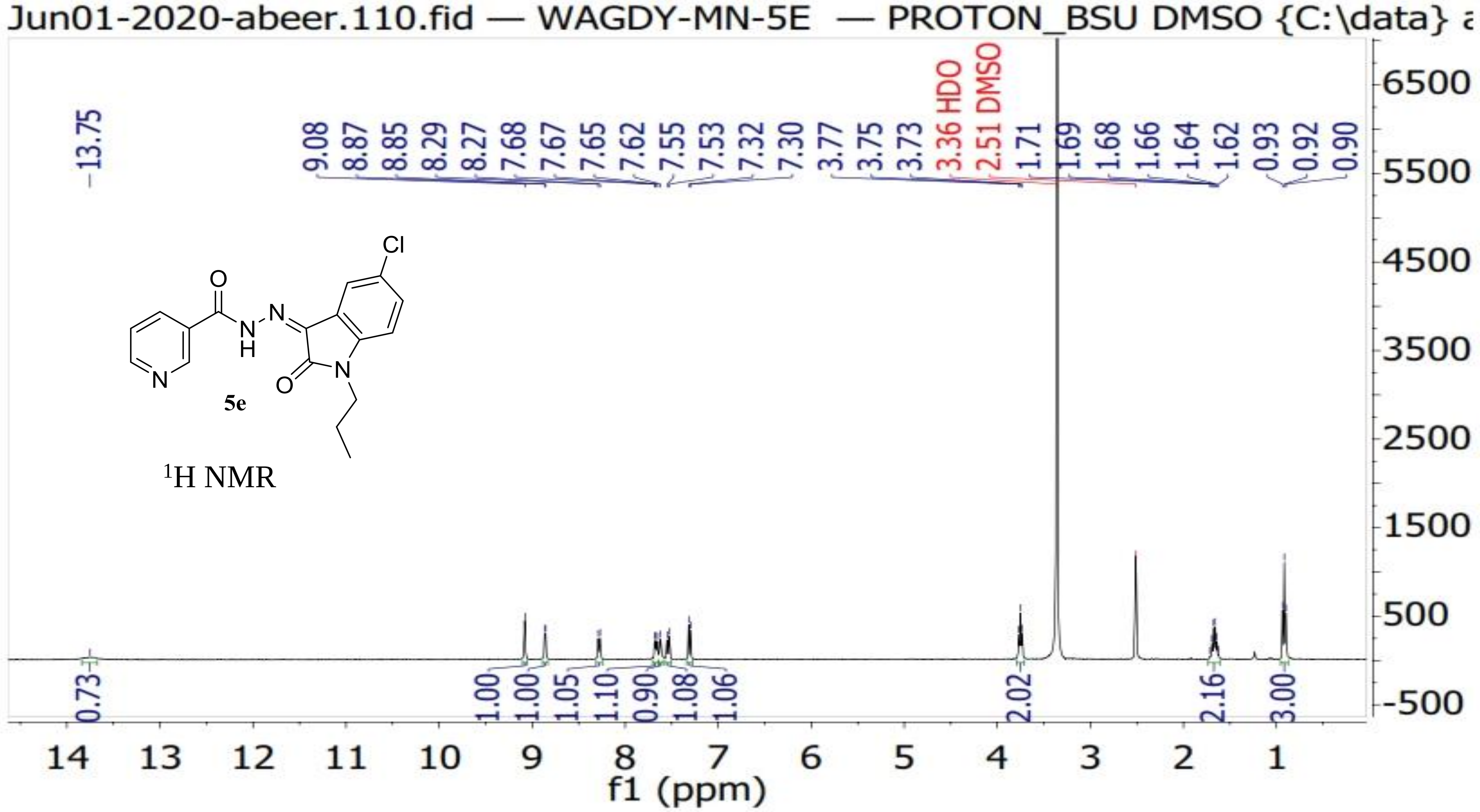
Jun01-2020-abeer.110.fid — WAGDY-MN-5E — PROTON_BSU DMSO {C:\data} a
-13.75
9.08 8.87 8.85 8.29 8.27 7.68 7.67 7.65 7.62 7.55 7.53 7.32 7.30 3.77 3.75 3.73 3.36 HDO 2.51 DMSO 1.71 1.69 1.68 1.66 1.64 1.62 0.93 0.92 0.90
0.73
1.00 1.00 1.05 1.10 0.90 1.08 1.06
2.02
2.16
3.00
6500
5500
4500
3500
2500
1500
500
-500
14 13 12 11 10 9 8 7 6 5 4 3 2 1
f1 (ppm)
Cl
O
N
N
H
N
O
N
5e
^{1}H NMR

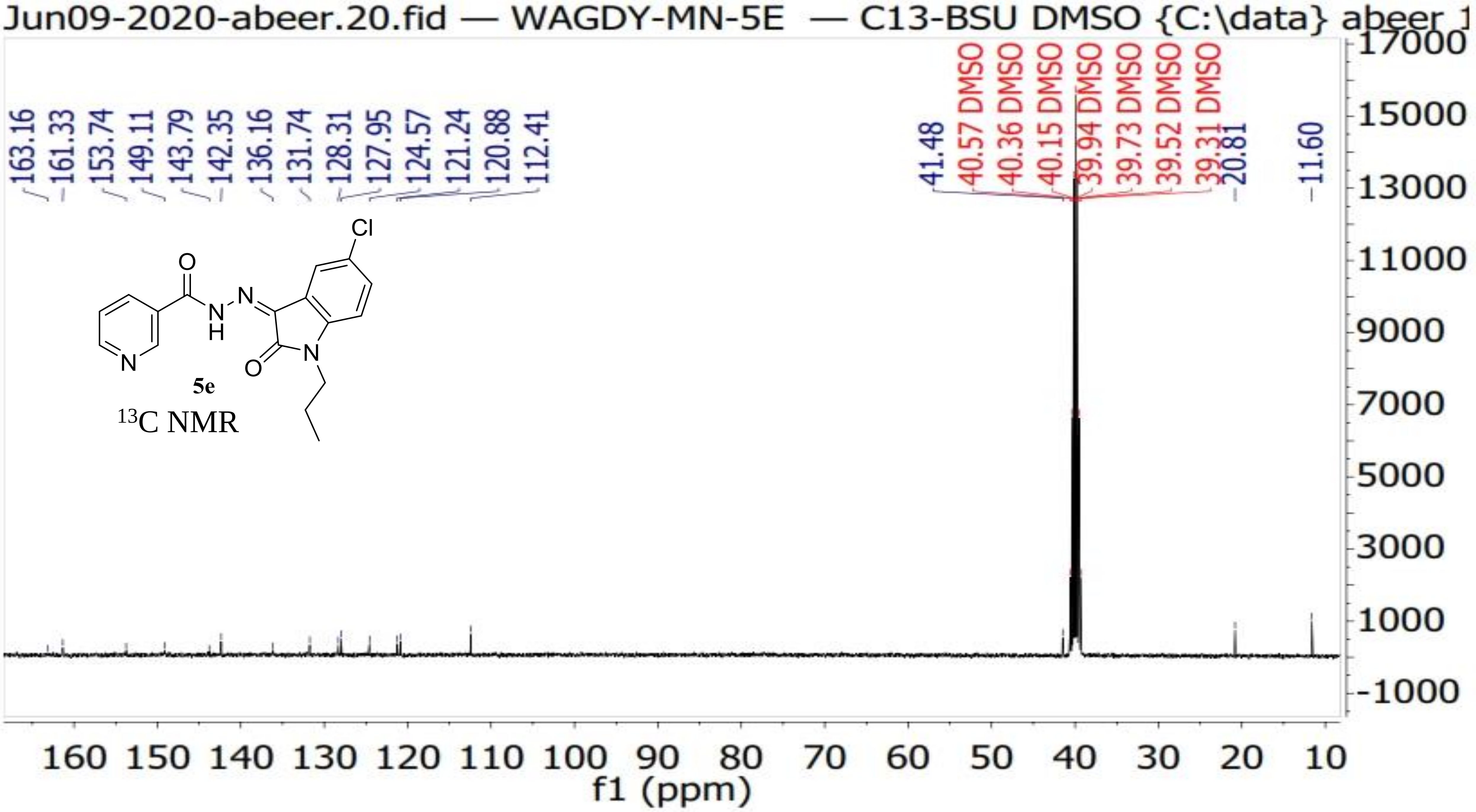
Jun09-2020-abeer.20.fid — WAGDY-MN-5E — C13-BSU DMSO {C:\data} abeer 1
163.16
161.33
153.74
149.11
143.79
142.35
136.16
131.74
128.31
127.95
124.57
121.24
120.88
112.41
41.48
40.57 DMSO
40.36 DMSO
40.15 DMSO
39.94 DMSO
39.73 DMSO
39.52 DMSO
39.31 DMSO
20.81
11.60
Cl
O
N
H
N
O
N
N
5e
^{13}C NMR
f1 (ppm)

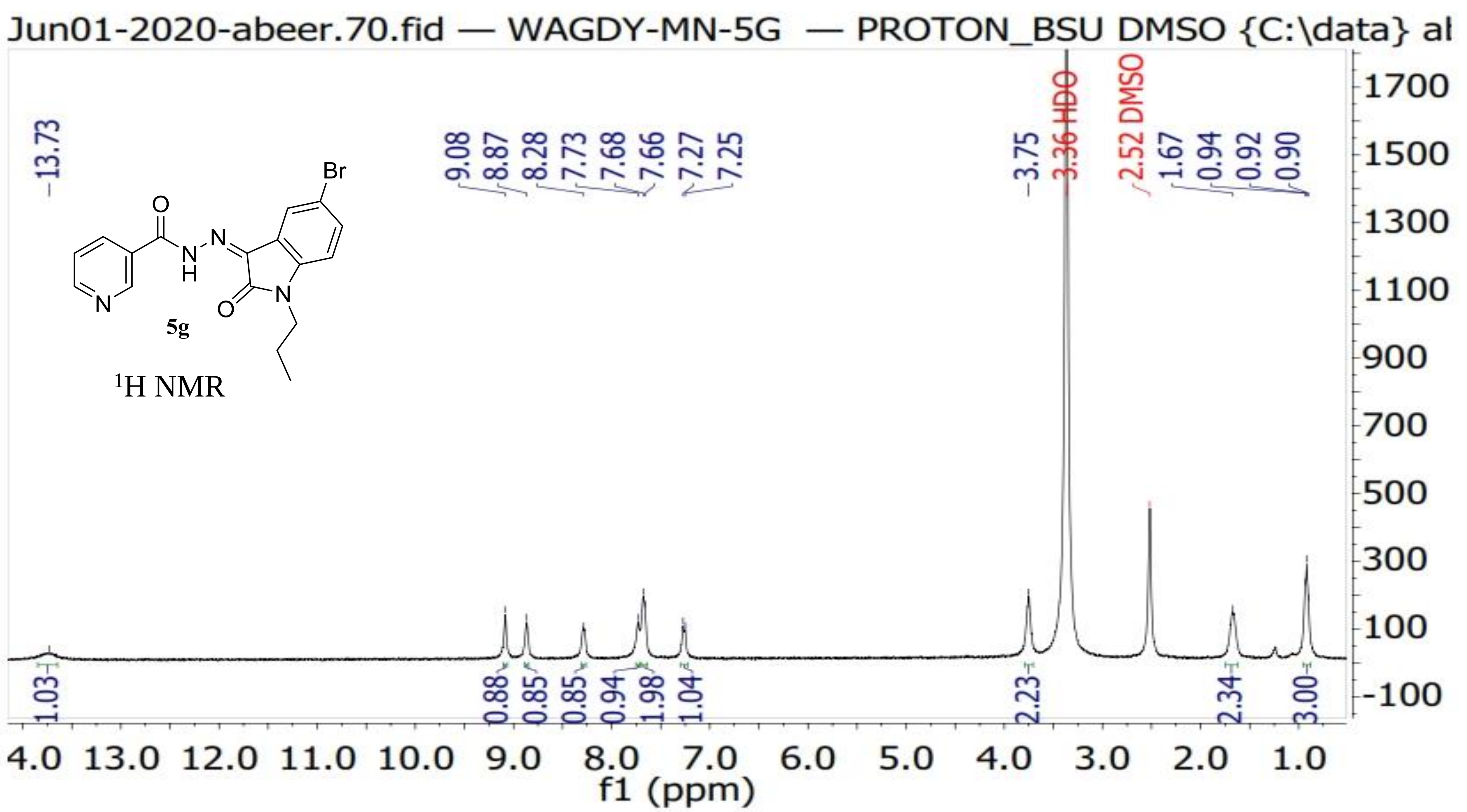
Jun01-2020-abeer.70.fid — WAGDY-MN-5G — PROTON_BSU DMSO {C:\data} al
13.73
9.08
8.87
8.28
7.73
7.68
7.66
7.27
7.25
3.75
3.36 HDO
2.52 DMSO
1.67
0.94
0.92
0.90
Br
O
N
N
H
N
O
N
5g
1H NMR
1.03
0.88
0.85
0.85
0.94
1.98
1.04
2.23
2.34
3.00
1700
1500
1300
1100
900
700
500
300
100
-100
4.0
13.0
12.0
11.0
10.0
9.0
8.0
7.0
6.0
5.0
4.0
3.0
2.0
1.0
f1 (ppm)

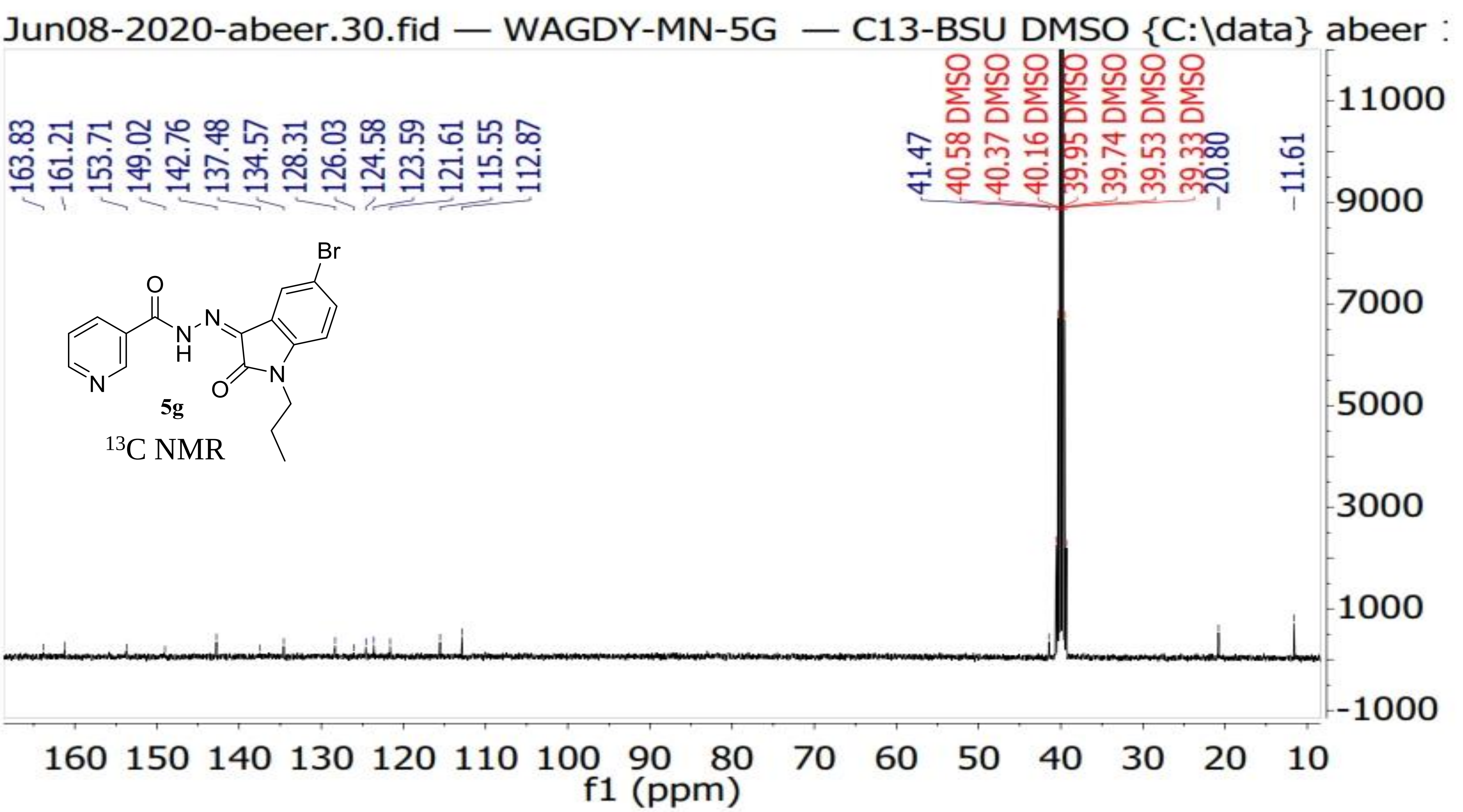
Jun08-2020-abeer.30.fid — WAGDY-MN-5G — C13-BSU DMSO {C:\data} abeer
163.83
161.21
153.71
149.02
142.76
137.48
134.57
128.31
126.03
124.58
123.59
121.61
115.55
112.87
41.47
40.58 DMSO
40.37 DMSO
40.16 DMSO
39.95 DMSO
39.74 DMSO
39.53 DMSO
39.33 DMSO
20.80
11.61
Br
O
N
N
H
N
O
N
5g
13C NMR
11000
9000
7000
5000
3000
1000
-1000
160 150 140 130 120 110 100 90 80 70 60 50 40 30 20 10
f1 (ppm)

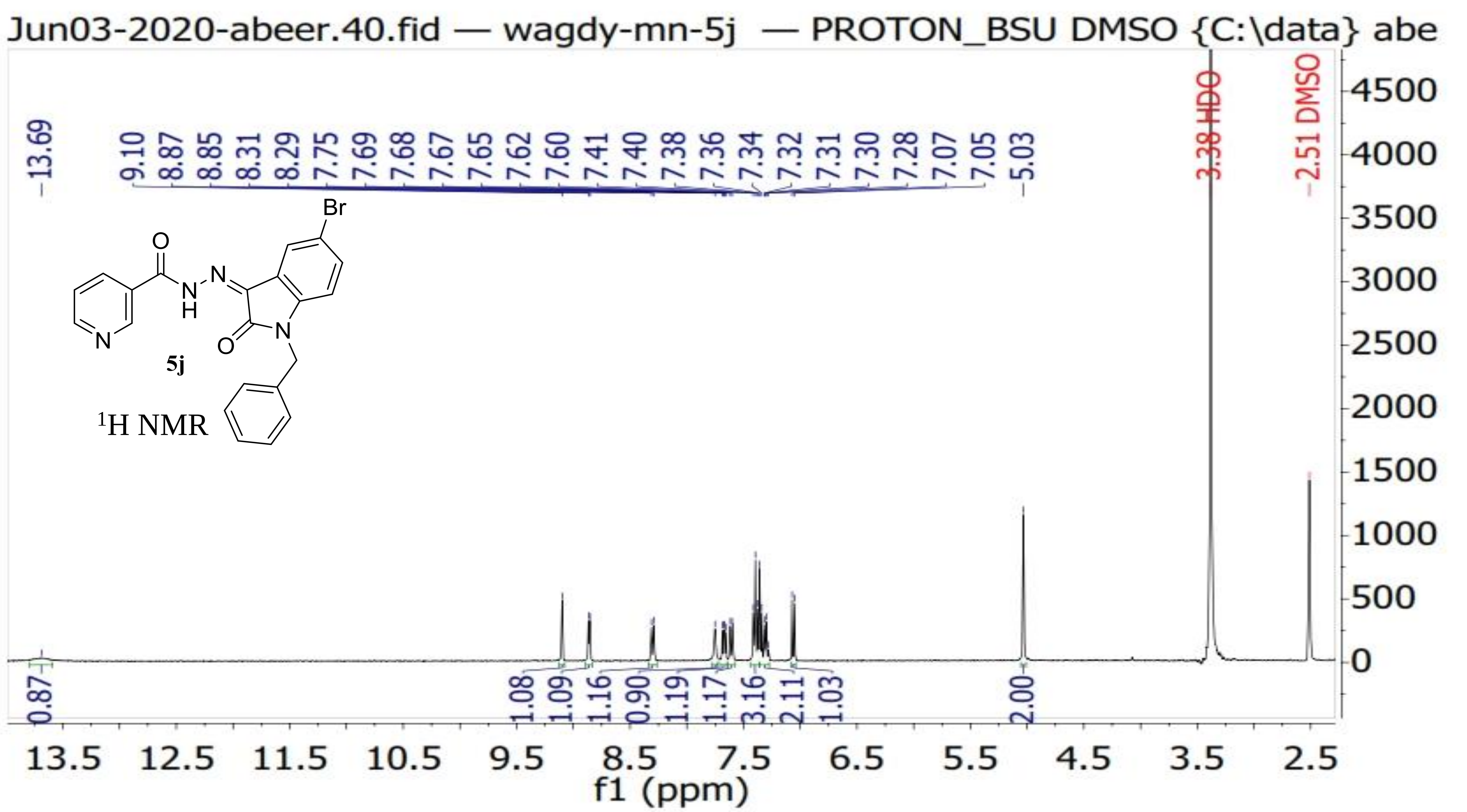
Jun03-2020-abeer.40.fid — wagdy-mn-5j — PROTON_BSU DMSO {C:\data} abe
13.69
9.10 8.87 8.85 8.31 8.29 7.75 7.69 7.68 7.67 7.65 7.62 7.60 7.41 7.40 7.38 7.36 7.34 7.32 7.31 7.30 7.28 7.07 7.05
5.03
3.38 HDO
2.51 DMSO
Br
O
N
N
H
N
O
N
5j
¹H NMR
0.87
1.08 1.09 1.16 0.90 1.19 1.17 3.16 2.11 1.03
2.00
4500
4000
3500
3000
2500
2000
1500
1000
500
0
13.5 12.5 11.5 10.5 9.5 8.5 7.5 6.5 5.5 4.5 3.5 2.5
f1 (ppm)

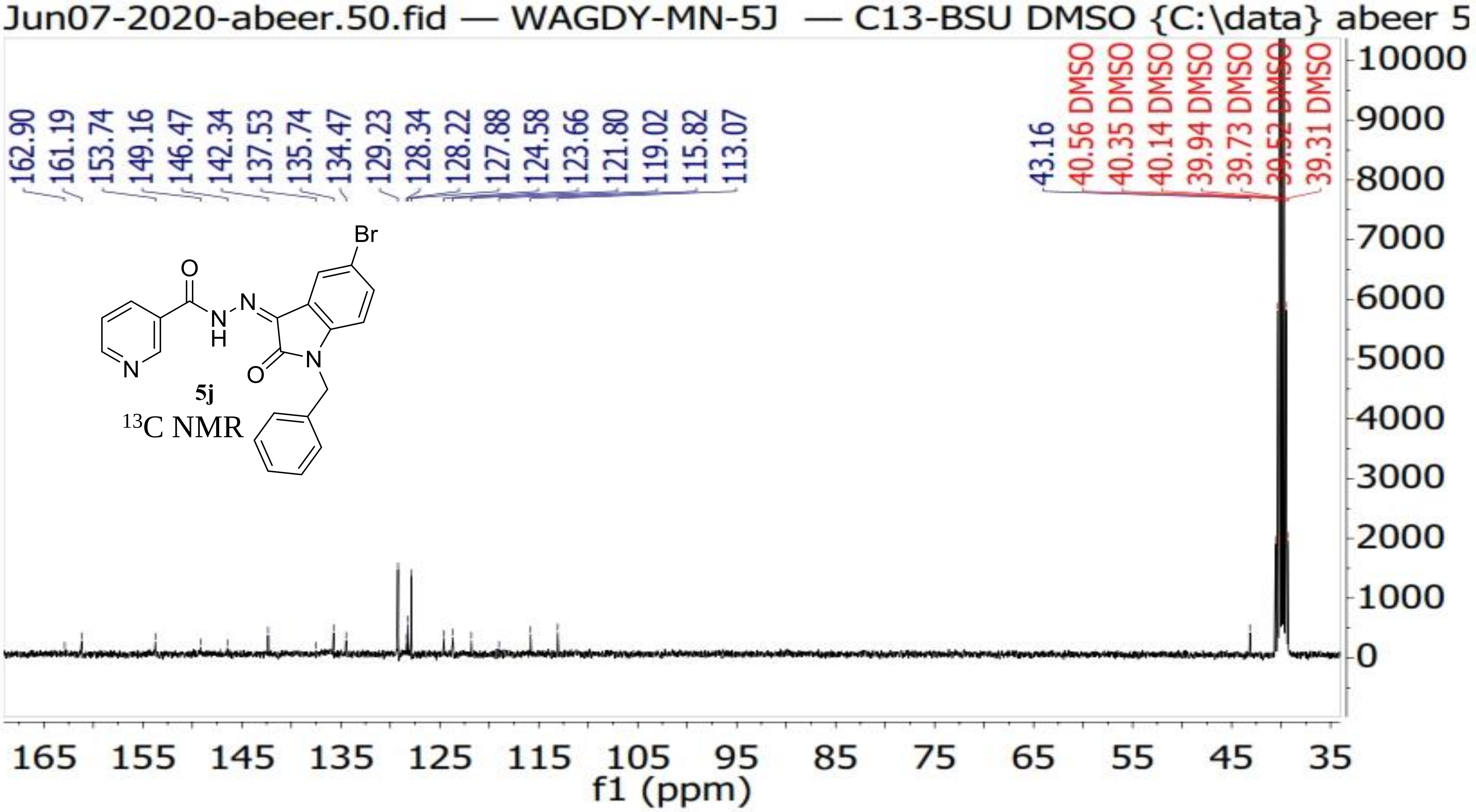
Jun07-2020-abeer.50.fid — WAGDY-MN-5J — C13-BSU DMSO {C:\data} abeer 5
162.90
161.19
153.74
149.16
146.47
142.34
137.53
135.74
134.47
129.23
128.34
128.22
127.88
124.58
123.66
121.80
119.02
115.82
113.07
43.16
40.56 DMSO
40.35 DMSO
40.14 DMSO
39.94 DMSO
39.73 DMSO
39.52 DMSO
39.31 DMSO
Br
O
N
N
H
N
O
N
5j
13C NMR
10000
9000
8000
7000
6000
5000
4000
3000
2000
1000
0
165
155
145
135
125
115
105
95
85
75
65
55
45
35
f1 (ppm)

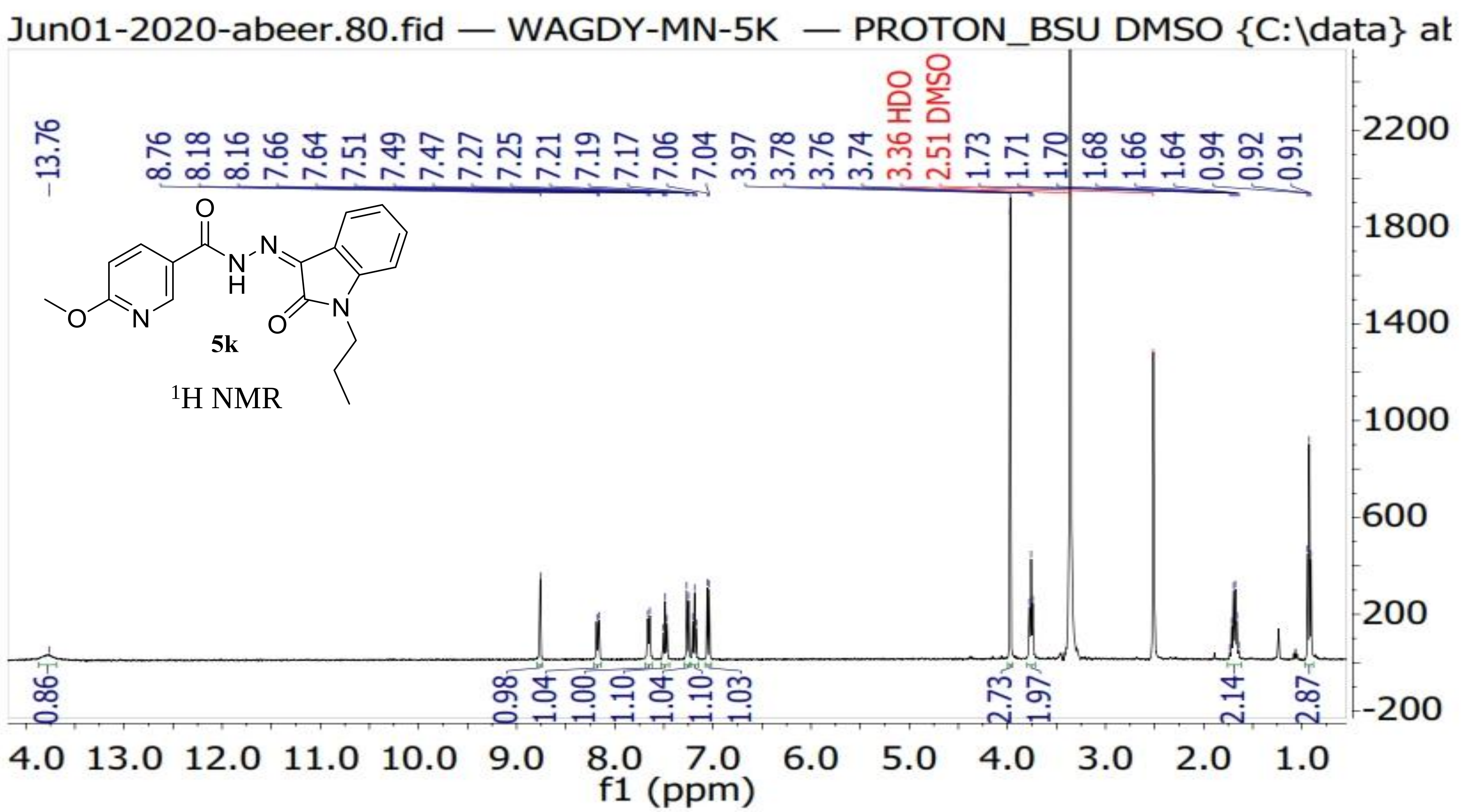

Jun01-2020-abeer.80.fid — WAGDY-MN-5K — PROTON_BSU DMSO {C:\data} al
13.76
8.76 8.18 8.16 7.66 7.64 7.51 7.49 7.47 7.27 7.25 7.21 7.19 7.17 7.06 7.04
3.97 3.78 3.76 3.74 3.36 HDO 2.51 DMSO 1.73 1.71 1.70 1.68 1.66 1.64 0.94 0.92 0.91
0.86 0.98 1.04 1.00 1.10 1.04 1.10 1.03 2.73 1.97 2.14 2.87
5k
1H NMR
2200
1800
1400
1000
600
200
-200
4.0 13.0 12.0 11.0 10.0 9.0 8.0 7.0 6.0 5.0 4.0 3.0 2.0 1.0
f1 (ppm)

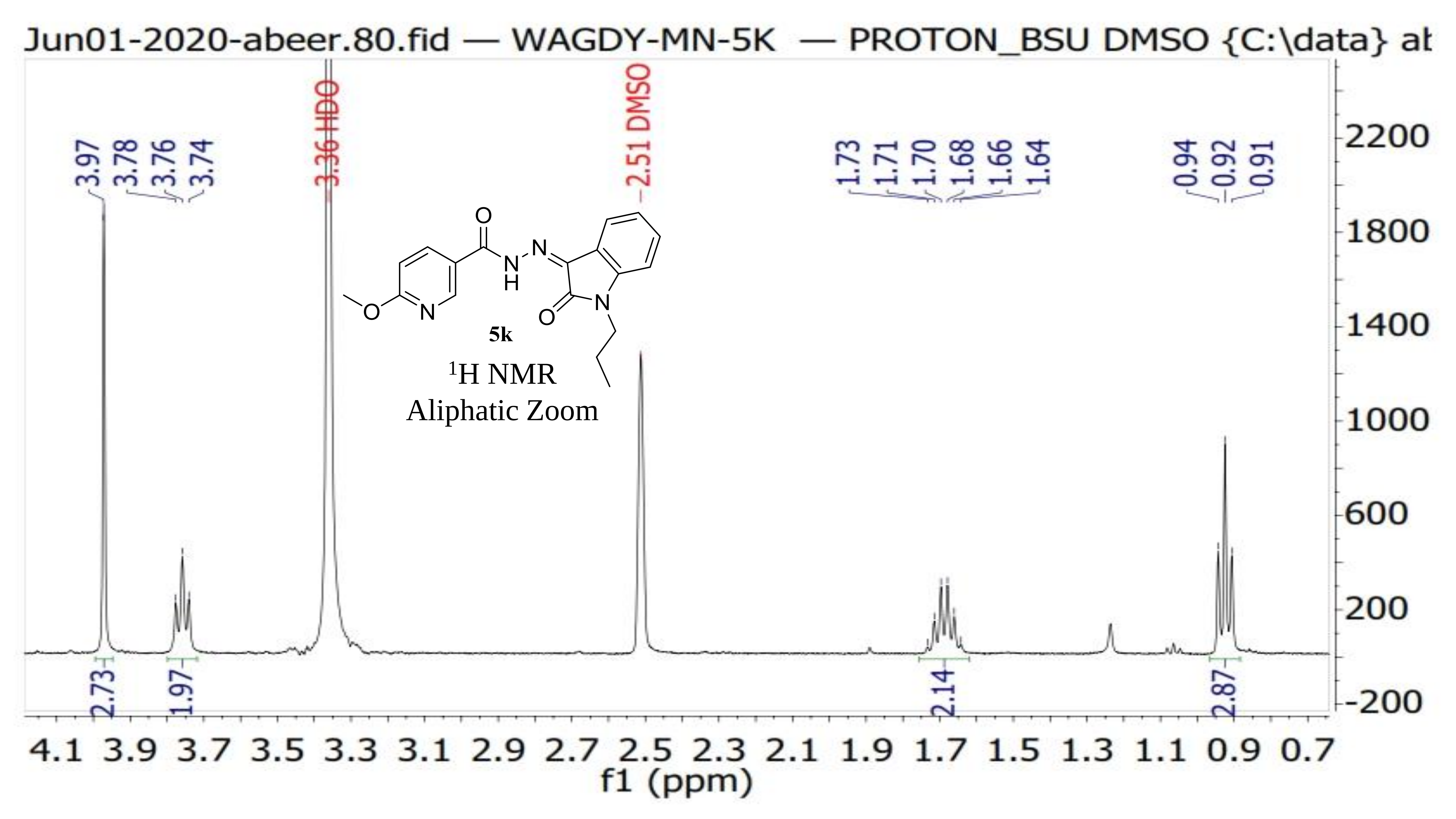
Jun01-2020-abeer.80.fid — WAGDY-MN-5K — PROTON_BSU DMSO {C:\data} ab
3.97
3.78
3.76
3.74
3.36 HDO
2.51 DMSO
1.73
1.71
1.70
1.68
1.66
1.64
0.94
0.92
0.91
5k
^{1}H NMR
Aliphatic Zoom
2.73
1.97
2.14
2.87
2200
1800
1400
1000
600
200
-200
4.1 3.9 3.7 3.5 3.3 3.1 2.9 2.7 2.5 2.3 2.1 1.9 1.7 1.5 1.3 1.1 0.9 0.7
f1 (ppm)

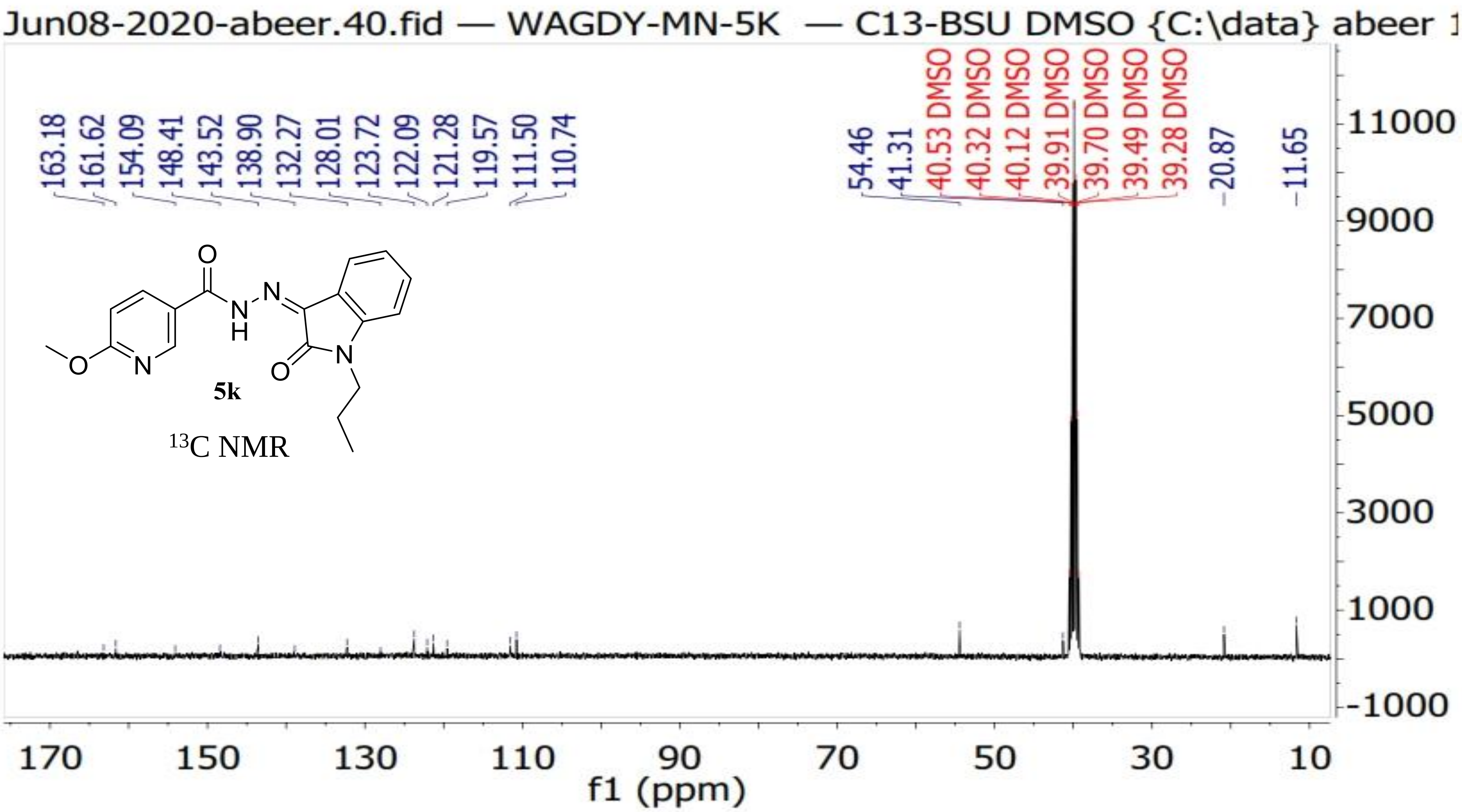

Jun08-2020-abeer.40.fid — WAGDY-MN-5K — C13-BSU DMSO {C:\data} abeer 1
163.18
161.62
154.09
148.41
143.52
138.90
132.27
128.01
123.72
122.09
121.28
119.57
111.50
110.74
54.46
41.31
40.53 DMSO
40.32 DMSO
40.12 DMSO
39.91 DMSO
39.70 DMSO
39.49 DMSO
39.28 DMSO
20.87
11.65
5k
13C NMR
11000
9000
7000
5000
3000
1000
-1000
170
150
130
110
90
70
50
30
10
f1 (ppm)

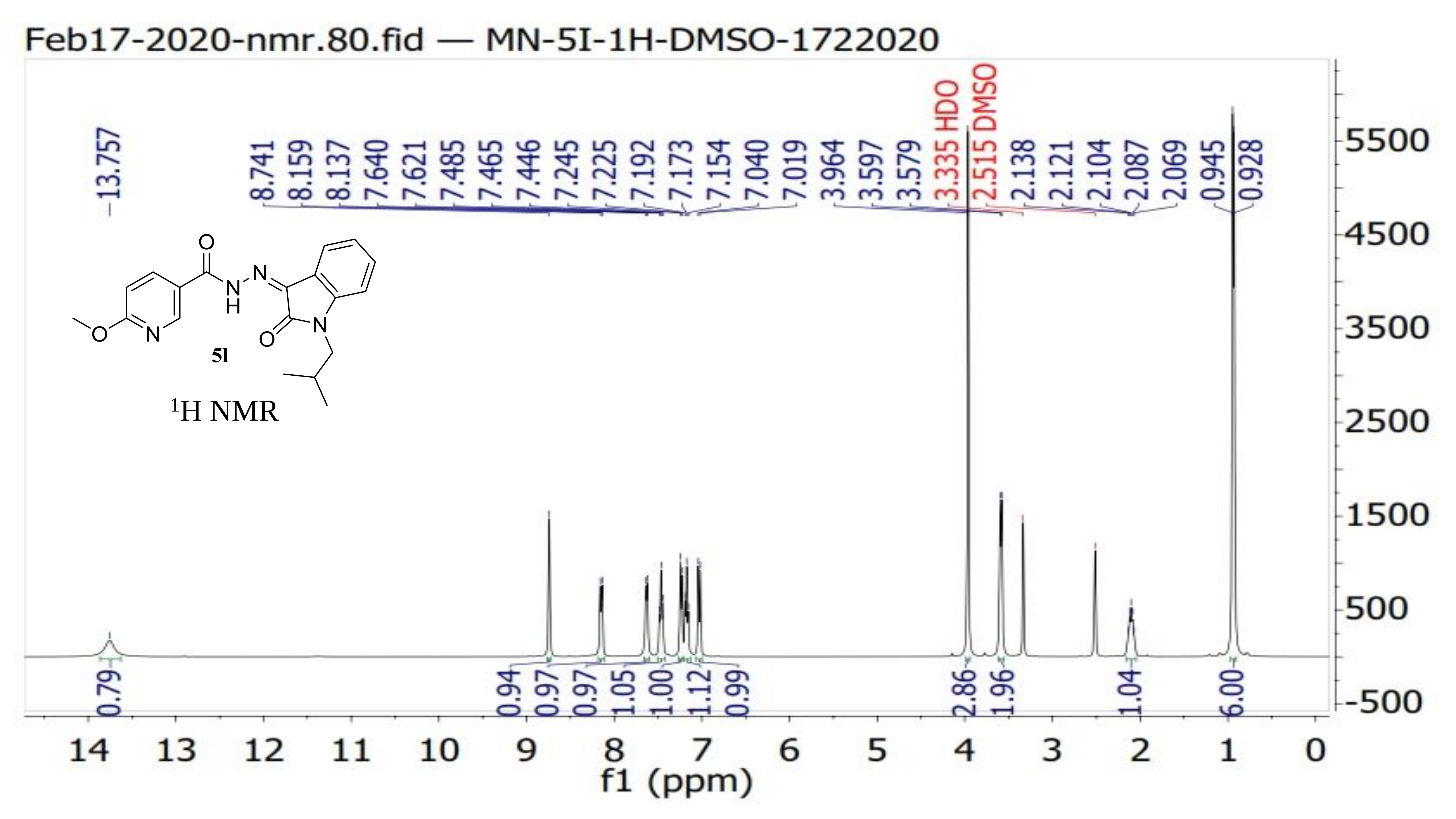
Feb17-2020-nmr.80.fid — MN-5I-1H-DMSO-1722020
13.757
8.741
8.159
8.137
7.640
7.621
7.485
7.465
7.446
7.245
7.225
7.192
7.173
7.154
7.040
7.019
3.964
3.597
3.579
3.335 HDO
2.515 DMSO
2.138
2.121
2.104
2.087
2.069
0.945
0.928
5l
1H NMR
0.79
0.94
0.97
0.97
1.05
1.00
1.12
0.99
2.86
1.96
1.04
6.00
f1 (ppm)

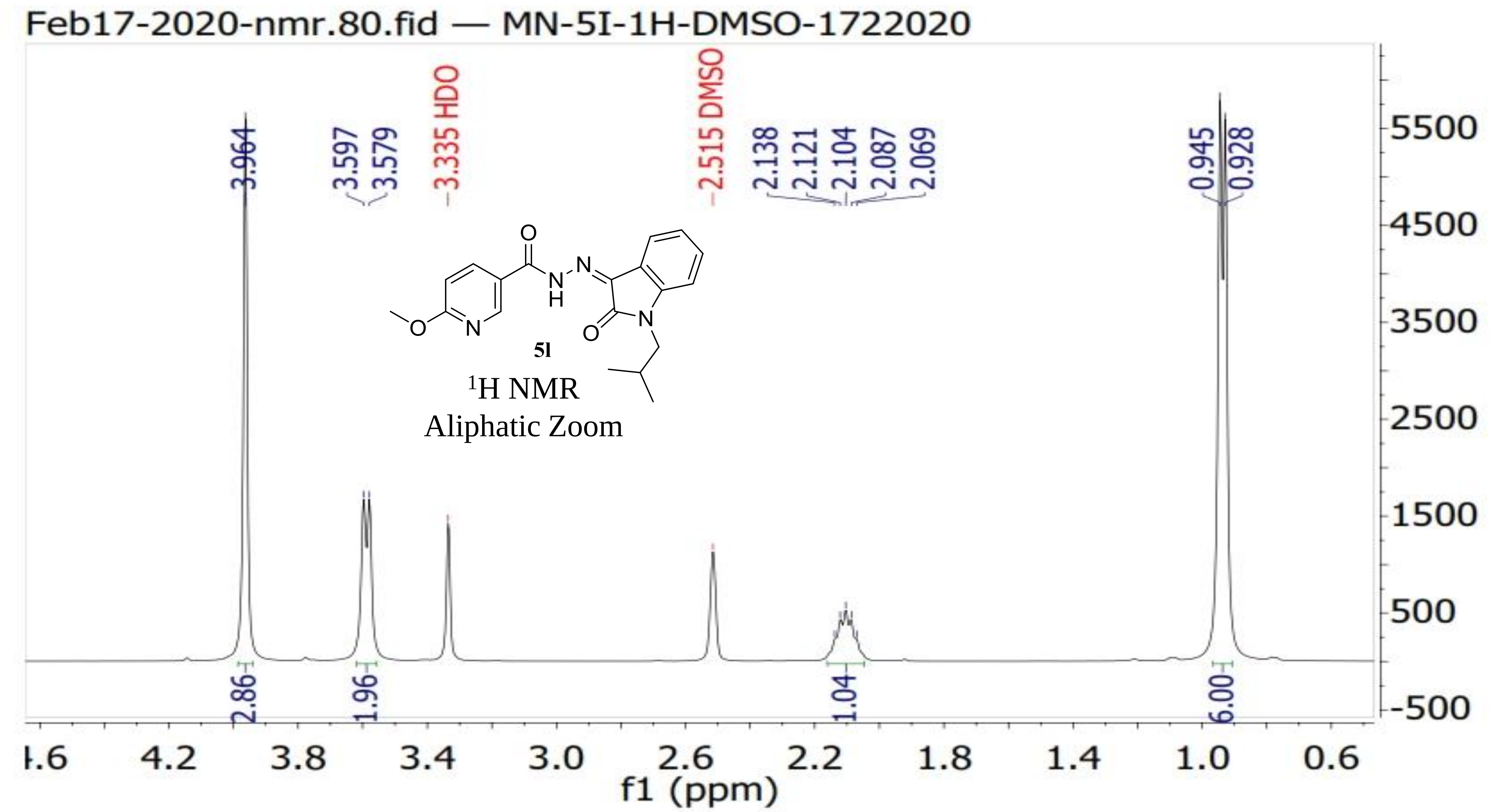
Feb17-2020-nmr.80.fid — MN-5I-1H-DMSO-1722020
3.964
3.597
3.579
3.335 HDO
2.515 DMSO
2.138
2.121
2.104
2.087
2.069
0.945
0.928
5l
1H NMR
Aliphatic Zoom
2.86
1.96
1.04
6.00
5500
4500
3500
2500
1500
500
-500
4.2
3.8
3.4
3.0
2.6
2.2
1.8
1.4
1.0
0.6
f1 (ppm)

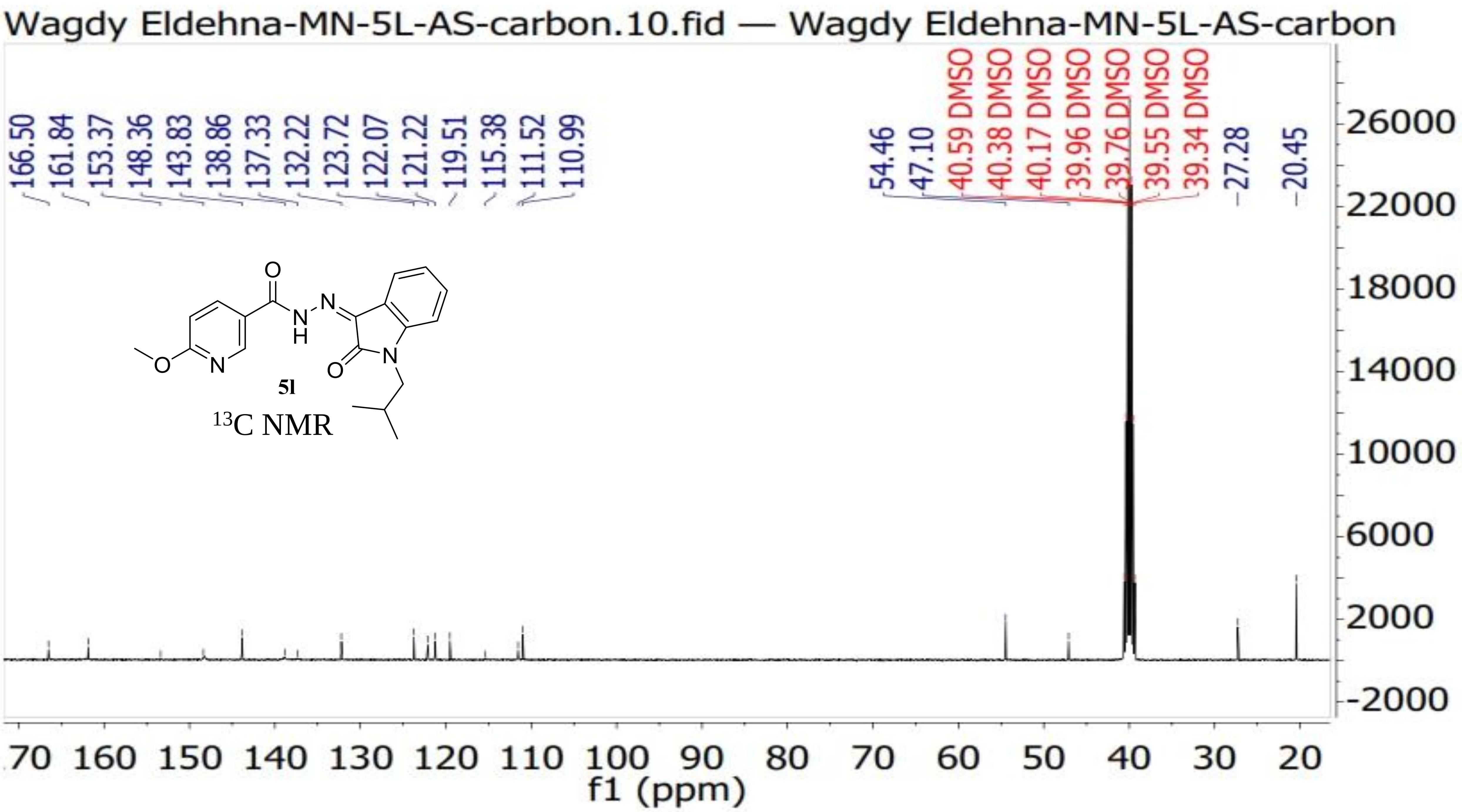
Wagdy Eldehna-MN-5L-AS-carbon.10.fid — Wagdy Eldehna-MN-5L-AS-carbon
166.50
161.84
153.37
148.36
143.83
138.86
137.33
132.22
123.72
122.07
121.22
119.51
115.38
111.52
110.99
54.46
47.10
40.59 DMSO
40.38 DMSO
40.17 DMSO
39.96 DMSO
39.76 DMSO
39.55 DMSO
39.34 DMSO
27.28
20.45
5l
13C NMR
f1 (ppm)

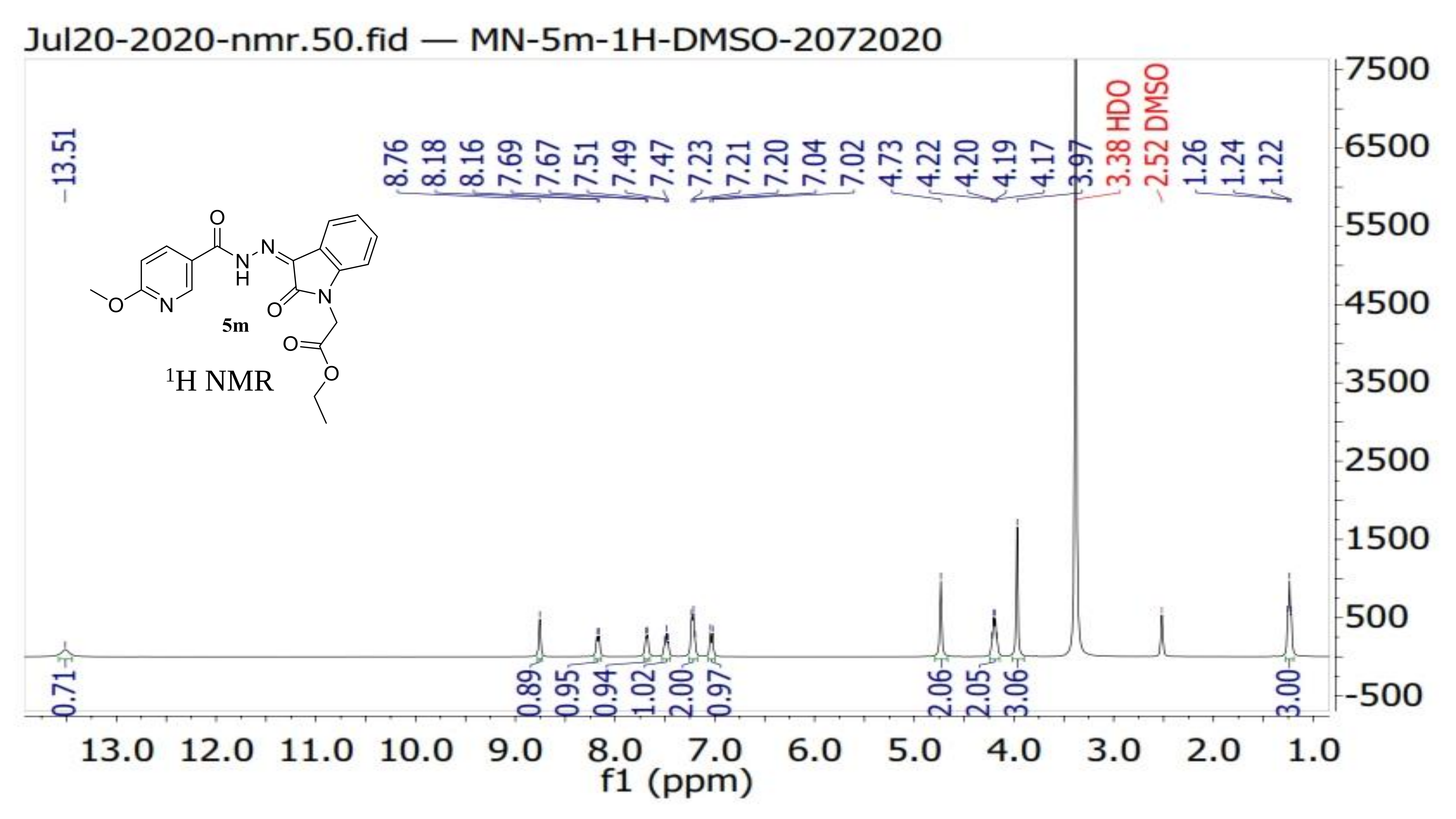
Jul20-2020-nmr.50.fid — MN-5m-1H-DMSO-2072020
13.51
8.76
8.18
8.16
7.69
7.67
7.51
7.49
7.47
7.23
7.21
7.20
7.04
7.02
4.73
4.22
4.20
4.19
4.17
3.97
3.38 HDO
2.52 DMSO
1.26
1.24
1.22
5m
1H NMR
0.71
0.89
0.95
0.94
1.02
2.00
0.97
2.06
2.05
3.06
3.00
f1 (ppm)

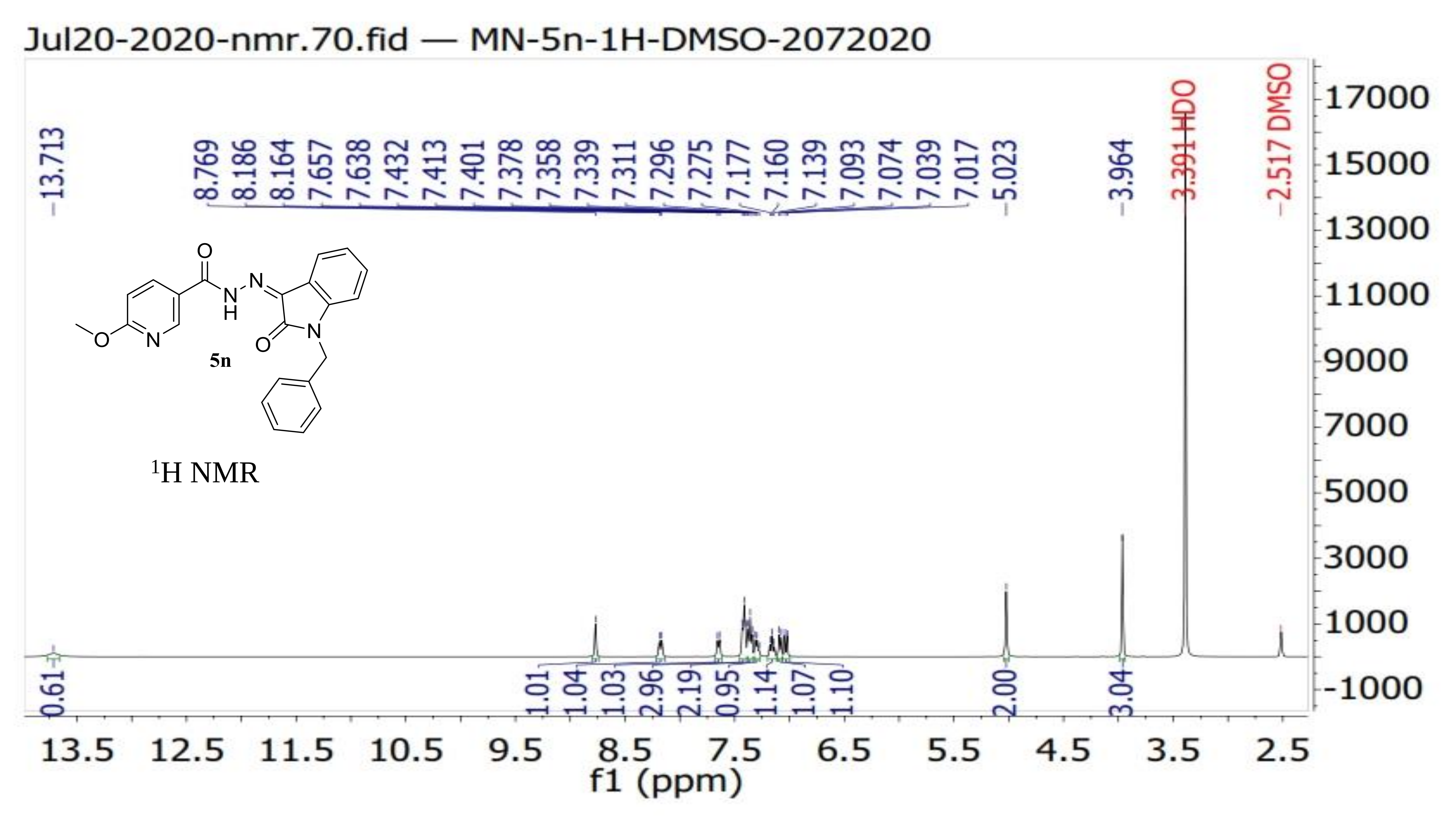
Jul20-2020-nmr.70.fid — MN-5n-1H-DMSO-2072020
13.713
8.769
8.186
8.164
7.657
7.638
7.432
7.413
7.401
7.378
7.358
7.339
7.311
7.296
7.275
7.177
7.160
7.139
7.093
7.074
7.039
7.017
5.023
3.964
3.391 HDO
2.517 DMSO
5n
1H NMR
0.61
1.01
1.04
1.03
2.96
2.19
0.95
1.14
1.07
1.10
2.00
3.04
f1 (ppm)

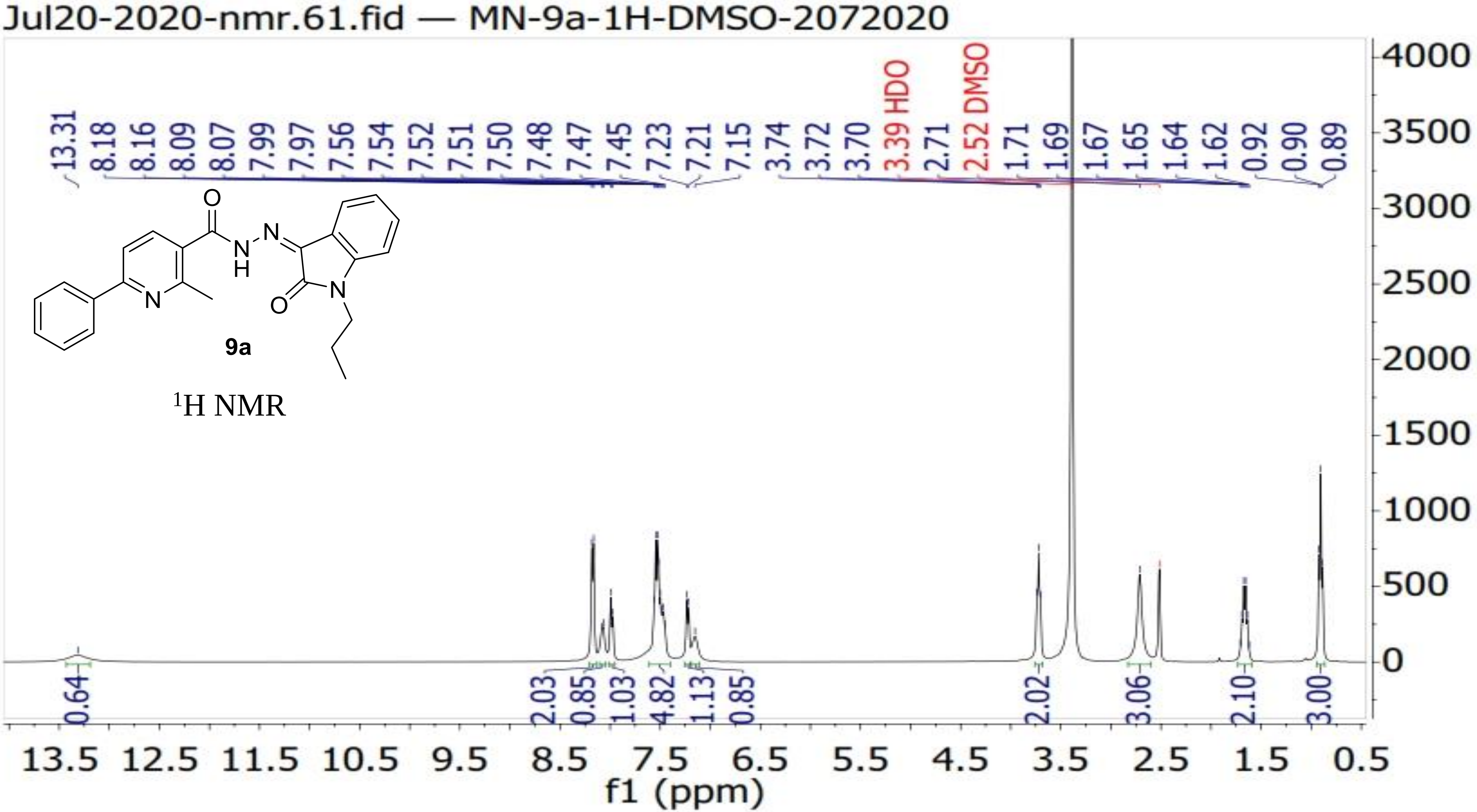
Jul20-2020-nmr.61.fid — MN-9a-1H-DMSO-2072020
13.31 8.18 8.16 8.09 8.07 7.99 7.97 7.56 7.54 7.52 7.51 7.50 7.48 7.47 7.45 7.23 7.21 7.15 3.74 3.72 3.70 3.39 HDO 2.71 2.52 DMSO 1.71 1.69 1.67 1.65 1.64 1.62 0.92 0.90 0.89
9a
1H NMR
0.64 2.03 0.85 1.03 4.82 1.13 0.85 2.02 3.06 2.10 3.00
f1 (ppm)

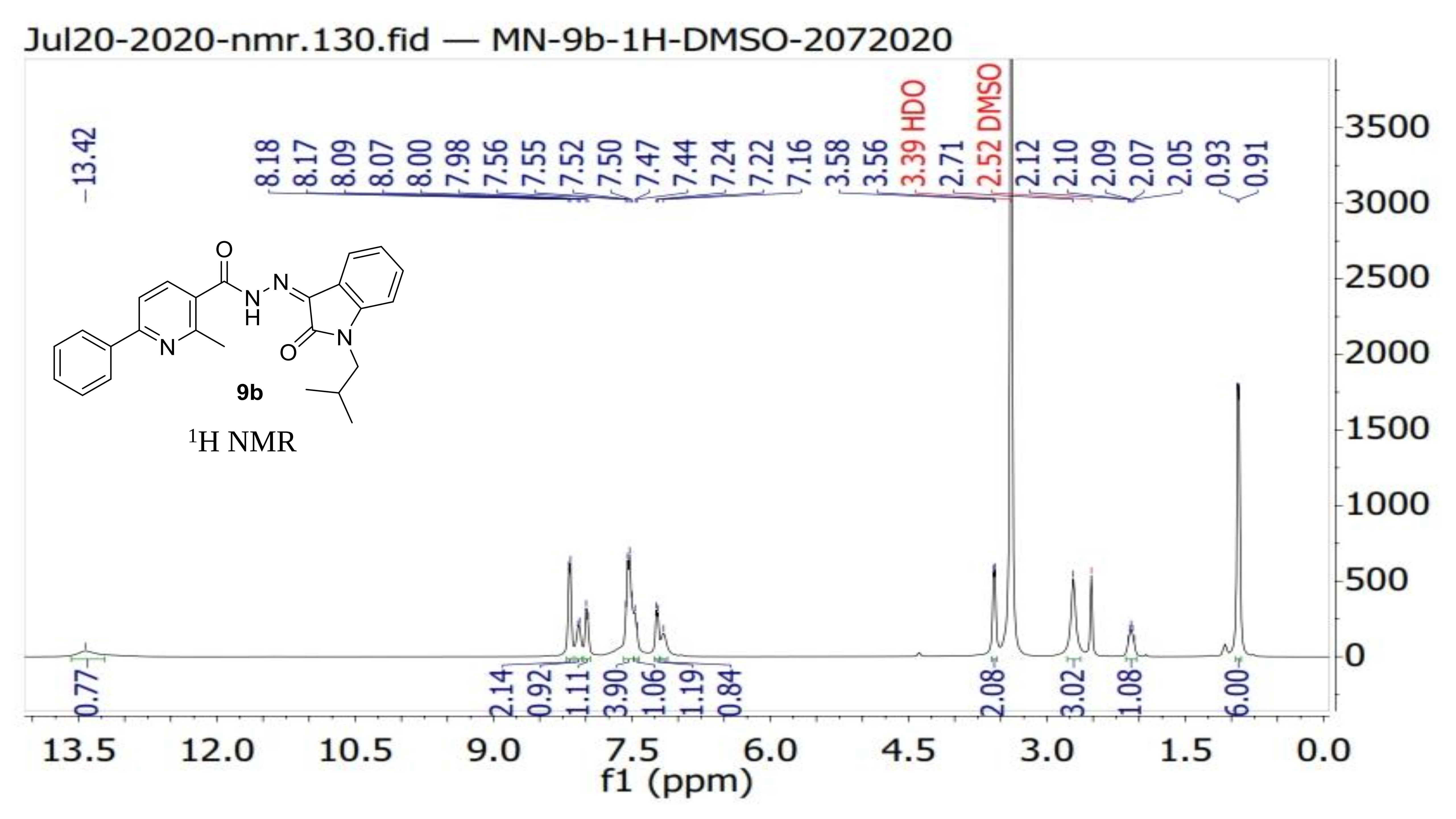
Jul20-2020-nmr.130.fid — MN-9b-1H-DMSO-2072020
13.42
8.18 8.17 8.09 8.07 8.00 7.98 7.56 7.55 7.52 7.50 7.47 7.44 7.24 7.22 7.16 3.58 3.56 3.39 HDO 2.71 2.52 DMSO 2.12 2.10 2.09 2.07 2.05 0.93 0.91
9b
1H NMR
0.77 2.14 0.92 1.11 3.90 1.06 1.19 0.84 2.08 3.02 1.08 6.00
f1 (ppm)

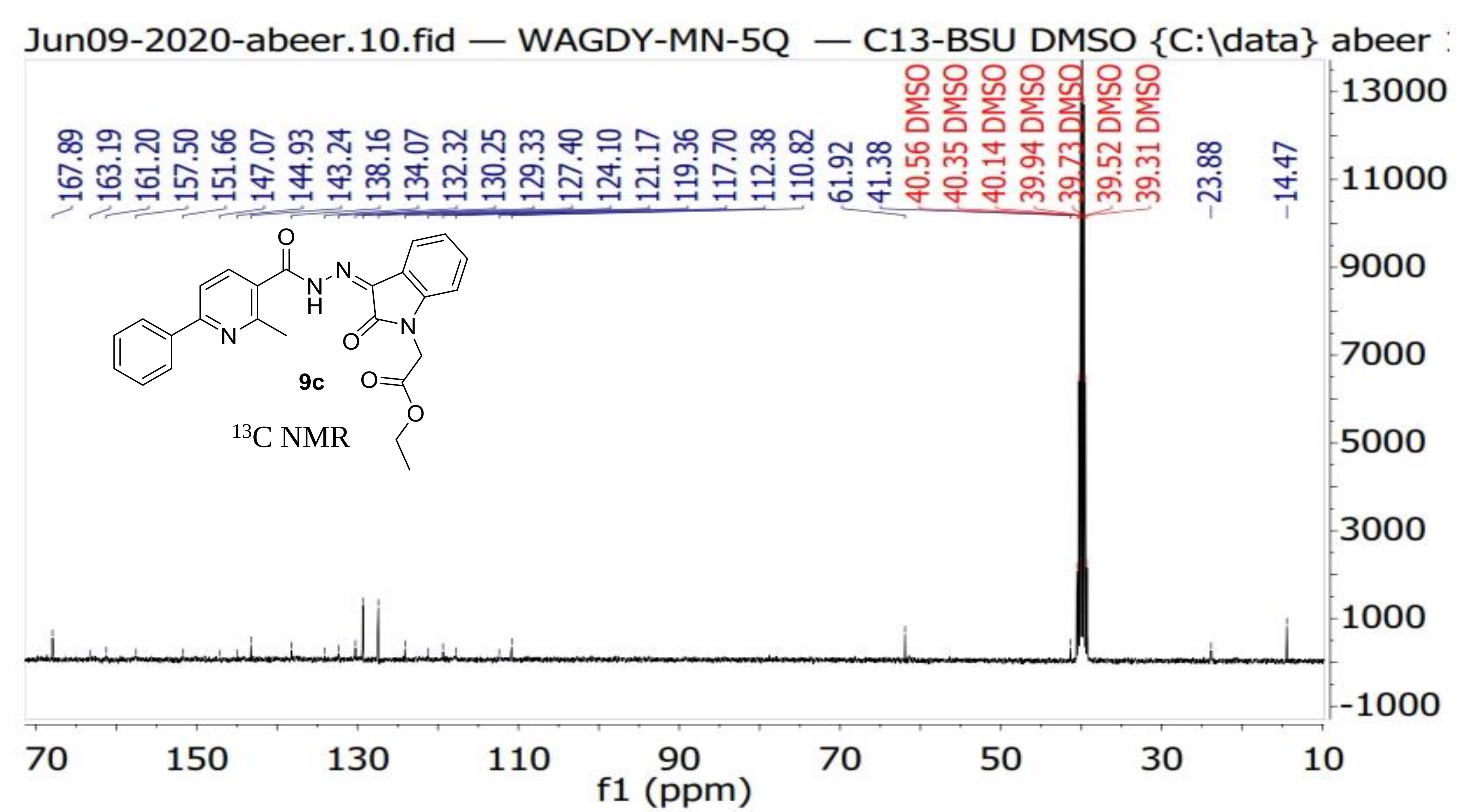
Jun09-2020-abeer.10.fid — WAGDY-MN-5Q — C13-BSU DMSO {C:\data} abeer
167.89
163.19
161.20
157.50
151.66
147.07
144.93
143.24
138.16
134.07
132.32
130.25
129.33
127.40
124.10
121.17
119.36
117.70
112.38
110.82
61.92
41.38
40.56 DMSO
40.35 DMSO
40.14 DMSO
39.94 DMSO
39.73 DMSO
39.52 DMSO
39.31 DMSO
23.88
14.47
9c
13C NMR
f1 (ppm)

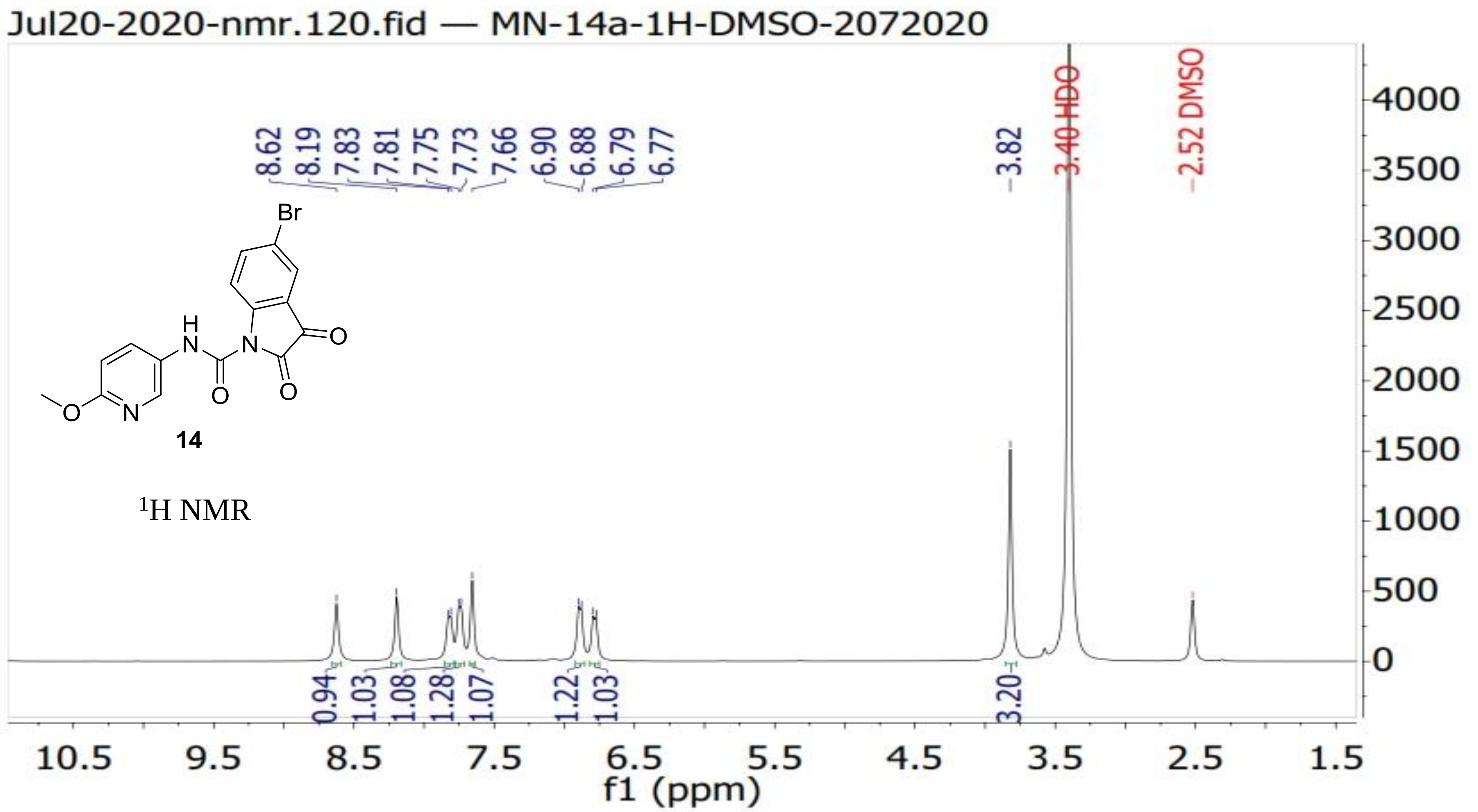

Jul20-2020-nmr.120.fid — MN-14a-1H-DMSO-2072020
8.62 8.19 7.83 7.81 7.75 7.73 7.66 6.90 6.88 6.79 6.77
3.82
3.40 HDO
2.52 DMSO
Br
H
N
N
O
O
O
O
N
14
^{1}H NMR
0.94 1.03 1.08 1.28 1.07 1.22 1.03 3.20
4000 3500 3000 2500 2000 1500 1000 500 0
10.5 9.5 8.5 7.5 6.5 5.5 4.5 3.5 2.5 1.5
f1 (ppm)
